# The mechanoreceptor DEG‐1 regulates cold tolerance in *Caenorhabditis elegans*

**DOI:** 10.15252/embr.201948671

**Published:** 2020-02-03

**Authors:** Natsune Takagaki, Akane Ohta, Kohei Ohnishi, Akira Kawanabe, Yohei Minakuchi, Atsushi Toyoda, Yuichiro Fujiwara, Atsushi Kuhara

**Affiliations:** ^1^ Graduate School of Natural Science Konan University Kobe Japan; ^2^ Institute for Integrative Neurobiology Konan University Kobe Japan; ^3^ Faculty of Science and Engineering Konan University Kobe Japan; ^4^ Laboratory of Molecular Physiology & Biophysics Faculty of Medicine Kagawa University Kagawa Japan; ^5^ Advanced Genomics Center National Institute of Genetics Mishima Shizuoka Japan; ^6^ Comparative Genomics Laboratory National Institute of Genetics Mishima Shizuoka Japan; ^7^ AMED‐PRIME Japan Agency for Medical Research and Development Tokyo Japan

**Keywords:** *Caenorhabditis elegans*, cold tolerance, DEG/ENaC channel, DEG‐1, mechanoreceptor, temperature sensation, Membrane & Intracellular Transport, Neuroscience

## Abstract

*Caenorhabditis elegans* mechanoreceptors located in ASG sensory neurons have been found to sense ambient temperature, which is a key trait for animal survival. Here, we show that experimental loss of xanthine dehydrogenase (XDH‐1) function in AIN and AVJ interneurons results in reduced cold tolerance and atypical neuronal response to changes in temperature. These interneurons connect with upstream neurons such as the mechanoreceptor‐expressing ASG. Ca^2+^ imaging revealed that ASG neurons respond to warm temperature via the mechanoreceptor DEG‐1, a degenerin/epithelial Na^+^ channel (DEG/ENaC), which in turn affects downstream AIN and AVJ circuits. Ectopic expression of DEG‐1 in the ASE gustatory neuron results in the acquisition of warm sensitivity, while electrophysiological analysis revealed that DEG‐1 and human MDEG1 were involved in warm sensation. Taken together, these results suggest that cold tolerance is regulated by mechanoreceptor‐mediated circuit calculation.

## Graphical abstract

A DEG/ENaC‐type mechanoreceptor‐mediated neural circuit regulates cold tolerance in *Caenorhabditis elegans*.

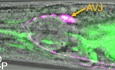

## Introduction

Temperature tolerance and acclimation are essential for all organisms, with such tolerance being controlled by multiple components such as the nervous system and muscles. *Caenorhabditis elegans* is an ideal model for studying the neural circuitry underlying cold tolerance given its simple nervous system composed of only 302 neurons, whose connections are entirely known, as well as the range of well‐studied molecular and genetic approaches currently available 1, 2. *Caenorhabditis elegans* mutants have also been extensively used to identify key genes and determine the specific neurons at which they exert their effects 3. Finally, *C. elegans* temperature response has been analyzed with respect to many phenomena, including dauer larva formation 3, thermotactic behavior 4, and cold tolerance 5, 6, 7, 8.

**Figure 1:**
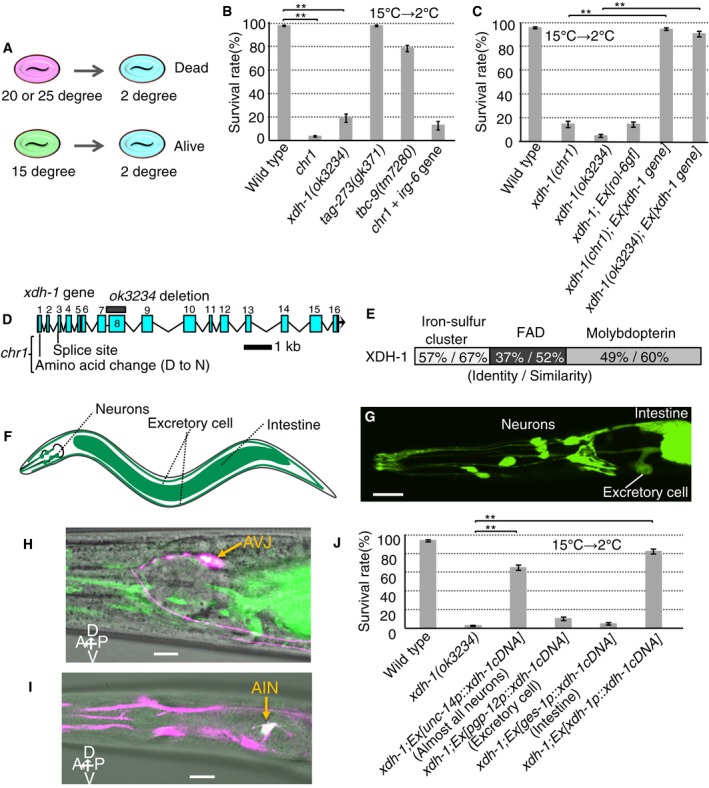
Neuronal XDH‐1 regulates cold tolerance Schematic of cold tolerance. Worms cultivated at 20°C or 25°C do not survive at 2°C, but those cultivated at 15°C do.
*xdh‐1* exhibits abnormal cold tolerance (number of assays ≥ 10).Transgenic rescue of *xdh‐1* mutants expressing wild‐type *xdh‐1* gene fused with GFP (number of assays ≥ 11).Exons of *xdh‐1* gene are boxed and numbered. *chr1* and *ok3234* mutations are shown.The amino acid identity and similarity between XDH‐1 and human XDH for each domain.Schematic diagram of expression pattern (green).
*xdh‐1p::gfp* expression in neurons, intestine, and excretory cells. Scale bar: 10 μm.Wild type expressing *xdh‐1p*::*xdh‐1 cDNA::gfp* (green) and *hlh‐34p(AVJp)::dsRedm* (magenta). Both are expressed in AVJ neuron (white). Scale bar: 10 μm.Wild type expressing *xdh‐1p::dsRedm* (magenta) and *inx‐17p(AINp)::yfp* (green). Both are expressed in AIN neuron (white). Scale bar: 10 μm.
*xdh‐1* abnormality was rescued by expressing *xdh‐1 cDNA* in neurons (number of assays ≥ 9). A part of data from wild type and *xdh‐1; Ex[unc‐14p::xdh‐1 cDNA]* are the same as those in Fig 2A, given that the experiments were conducted simultaneously.Data information: In (B, C, and J), the error bars indicate SEM. (B) **P < 0.01 (Dunnett's test). (C, J) **P < 0.01 (Tukey–Kramer).Source data are available online for this figure.

Taken together, the literature suggests that *C. elegans* possesses an adaptive mechanism to tolerate cold external environments. For example, wild‐type worms grown at 15°C can survive at a temperature of 2°C, whereas those grown at 20°C or 25°C cannot (Fig 1A) 5, 7, 8. Cold tolerance in nematodes is a process that involves a number of tissues/cells, including the bilateral pairs of specialized sensory neurons, intestinal cells, sperm, and muscle cells 5, 7, 8. In terms of sequence and site, the process begins when temperature is detected by the ASJ and ADL sensory neurons located in the head 5, 8. Next, insulin is released from the ASJ and binds to insulin receptors in the intestine and nervous tissue 8, which initiates steroid hormonal signaling to the sperm. Sperm in turn modulates ASJ neuronal activity in a feedback‐like manner 5. Genes are later expressed that ultimately modify bodily lipid composition 9, which is considered to be central to cold tolerance. The ability of the body to exhibit cold tolerance is established during cultivation under ambient conditions, not under cold conditions themselves 5. However, to date, these mechanisms have been mainly described in terms of the negative regulation of cold tolerance, while overlooking the as‐yet‐unexplored positive regulation of cold tolerance.

The ability of animals to detect temperature was previously studied with a focus on transient receptor potential (TRP) channels. TRPV1, for example, is known to detect regions of high temperature, while TRPA1 detects regions of low temperature 10. Regarding TRP‐independent temperature detection pathways, G protein‐coupled receptor (GPCR)/rhodopsin in *Drosophila* may act as a temperature receptor able to modulate decision‐making behavior 11. Furthermore, receptor‐type guanylyl cyclases (rGCs) in the nematode worm *C. elegans* are thought to function as temperature receptors in the AFD temperature‐sensing neuron given that the ectopic expression of rGCs can confer temperature‐dependent responses to heterologous cells 12. However, other temperature‐sensing mechanisms are thought to function in the detection of temperature in animals.

The degenerin/epithelial Na^+^ channel (DEG/ENaC) proteins comprise a diverse family of Na^+^ ion channels 13, 14, 15 involved in various cellular events such as mechanosensation 13, 16, sour/salt tastes 17, 18, 19, learning, memory, and synaptic plasticity 20, 21, 22, 23, 24. In mammals, the DEG/ENaC channel MDEG is abundantly expressed in the brain 14, while the *C. elegans* homologue DEG‐1 is expressed in association with multiple mechanosensory neurons 13, 25, 26. Although a decrease in temperature results in a change to the Na^+^ potential across MDEG 27, it remains unknown whether DEG/ENaCs are directly involved in the process of sensing temperature.

Xanthine dehydrogenases (XDHs) are widely distributed in all eukaryotic organisms and bacteria, and are predicted to play important roles such as in purine catabolism 28. In mammals, xanthine oxidoreductase (XOR) is present as two interconvertible forms: XDH and xanthine oxidase (XO). Moreover, both enzymes are active in the purine base salvage pathway. Notably, XDH converts hypoxanthine to xanthine, while also oxidizing xanthine to urate. Nicotinamide adenine dinucleotide (NADH) is also produced simultaneously during the final step of purine salvage 29.

Xanthine dehydrogenase is expressed in the liver and small intestine in mammals 30. A recent study suggested that XDH expression levels are associated with tumor growth. Elevated expression of XDH is associated with tumor infiltration as well as upregulated proinflammatory and immune‐related cytokine expression 31. XDH also served as a useful biological parameter in a pan‐cancer study 31, but its molecular function in neuronal or other cells remains largely unexplored given that it may be either reversibly or irreversibly converted to XO in mammals 32, 33. In contrast, since invertebrate XOR is present only as XDH 33, *C. elegans* is thought to be a useful model for studying XDH.

We found that *xdh‐1* mutants XDH knockout worms exhibit abnormal cold tolerance and that their normal function can be recovered by expressing *xdh‐1 cDNA* in both AIN and AVJ interneurons. *In vivo* Ca^2+^ imaging also revealed that XDH‐1 acts as a positive temperature signal regulator in AIN and as a negative one in AVJ and that warm sensation by ASG sensory neurons via the mechanoreceptor DEG‐1 affects the neural activity of both AIN and AVJ interneurons. Moreover, the ectopic expression of DEG‐1 in the ASE, a non‐warm‐sensitive chemosensory neuron, resulted in the acquisition of the ability to sense warm sensation. In addition, two‐electrode voltage‐clamp recording of *Xenopus* oocytes expressing DEG‐1 demonstrated thermoreceptor‐like behavior. These results together suggest that DEG‐1, a DEG/ENaC‐type mechanoreceptor, is sufficient to confer warm responses and is required for the neural circuit calculation of the positive regulation of cold tolerance.

## Results

### Xanthine dehydrogenase XDH‐1 regulates cold tolerance

To identify novel genes involved in cold tolerance, we isolated and analyzed a *chr1* mutation associated with decreased cold tolerance following cultivation at 15°C (Fig 1B and Appendix Fig S1A–G, see Appendix Supplementary Methods). A deep DNA sequencer and SNP analysis allowed the *chr1* mutation to be mapped to the region from 6.13 to 16.24 cM on chromosome IV, where four genes were found to exhibit major mutations (Appendix Fig S1D–F) (accession number DRA: 002599). We then evaluated cold tolerance in mutants for these genes (Fig 1B), with only *xdh‐1* mutants exhibiting markedly abnormal cold tolerance after cultivation at 15°C. Moreover, abnormal cold tolerance in both *chr1* and *xdh‐1* mutants was rescued by the expression of wild type *xdh‐1* (Fig 1C and D). These results together suggest that *xdh‐1* is the primary gene responsible for the observed phenotype of abnormal cold tolerance.

*xdh‐1* encodes the *C. elegans* homologue of human XDH (47% identity) (Fig 1E, and Appendix Fig S2A and B), and XDH itself contains iron–sulfur clusters, FAD, and molybdopterin domains (Fig 1E and Appendix Fig S2A–C). FAD is an NAD binding site, molybdopterin is a redox center, and XDH xanthine dehydrogenase in dimer form catalyzes the hydroxylation of xanthine as well as its subsequent conversion to uric acid (Appendix Fig S2C). Iron–sulfur cluster domains are strongly conserved throughout the animal kingdom (Appendix Fig S2A and B), and our *xdh‐1(chr1)* mutants possessed two point mutations in this domain: one in a conserved splicing acceptor site and the other in a nonconserved amino acid residue (Fig 1D and Appendix Fig S2B). Yet another allele, *ok3234*, contains a deletion mutation at an NAD binding site (Fig 1D and Appendix Fig S2B); we used this allele to conduct the following analysis.

### Intercellular reactive oxygen species concentrations and fatty acid composition in *xdh‐1* mutant

Xanthine dehydrogenase, an isoform of XOR, converts hypoxanthine to xanthine and also oxidizes xanthine to urate in the purine base salvage pathway 29. In mammals, XOR is present as two interconvertible forms: XDH and XO, both of which act enzymatically in the purine base salvage pathway. Uric acid functions as a potent antioxidant and protects against oxidative damage in vertebrates. To investigate whether defective XDH‐1 alters reactive oxygen species (ROS) concentrations in *xdh‐1* mutants, we used the fluorescent ROS indicator molecule 2′,7′‐dichlorodihydrofluorescein diacetate (H_2_DCF‐DA). Nonfluorescent H_2_DCF is converted to fluorescent 2′7′‐dichlorofluorescein (DCF) through interaction with intracellular ROS 34, 35, 36. We measured wild type, *xdh‐1(ok3234)*, and *daf‐2(e1370)* ROS concentrations, using the latter *daf‐2(e1370)* mutants as a control. In wild‐type worms, ROS concentrations gradually increased during the assay, while *xdh‐1(ok3234)* concentrations remained normal (Appendix Fig S3A). This suggests that *xdh‐1(ok3234)* mutants do not experience fluctuation in ROS concentration (Appendix Fig S3A). Similarly, we did not observe abnormal cold tolerance in *sod‐1*,* gst‐4*, or *bli‐3* ROS level mutants (Appendix Fig S3B).

In nematodes and other organisms, the proportion of fatty acids in the body is an important factor associated with cold tolerance 5, 7, 9. Measuring the fatty acid composition of total lipids in *xdh‐1* mutants, we found that this composition differed slightly between the wild type and *xdh‐1* mutants (Appendix Fig S3C and D). These results suggest that XDH‐1 may influence bodily fatty acid composition in wild‐type worms.

### Neuronal XDH‐1 function is required for cold tolerance

Cells expressing XDH‐1 located in head neurons including the AVJ and AIN neurons, the intestine, and excretory cells were analyzed by fluorescent protein expression driven by the *xdh‐1* promoter (Fig 1F–I and Appendix Fig S4A–C). To determine the minimum *xdh‐1* gene promoter length necessary for normal cold tolerance, we constructed a transgenic *xdh‐1* mutant strain expressing *xdh‐1 cDNA* fused with GFP driven by four different *xdh‐1* promoter lengths: 428, 952, 1,772, and 3,346 bp (Appendix Fig S4D and E). Abnormal *xdh‐1* mutant cold tolerance was rescued at all promoter lengths (Appendix Fig S4D). Although GFP fluorescence slightly decreased as promoter length decreased, the expression patterns were similar for all four promoter lengths (Appendix Fig S4E). This suggests that the 428 bp promoter region upstream of the *xdh‐1* start codon contains a region essential for the rescue of cold tolerance.

To identify the essential tissue(s) responsible for *xdh‐1*‐dependent cold tolerance, we then expressed *xdh‐1 cDNA* in specific tissues (Fig 1J and Appendix Table S1) and found that XDH‐1 expression in nearly all neurons restored abnormal cold tolerance (Fig 1J and Appendix Table S1; *xdh‐1; Ex[unc‐14p::xdh‐1 cDNA]*). *xdh‐1 cDNA* expression in intestine and excretory cells, however, did not rescue cold tolerance (Fig 1J and Appendix Table S1). This suggests that neuronal XDH‐1 activity is sufficient to maintain cold tolerance in *C. elegans*.

To determine whether neuronal XDH‐1‐dependent cold tolerance is established at the adult stage, we performed cold tolerance experiments using worms at adult and larval stages. We found that defective cold tolerance of *xdh‐1* mutants was severer at the adult stage than that at the larval one (Appendix Fig S4F). This implies that XDH‐1 may be mainly involved in the function of neurons in cold tolerance, rather than in its development. To identify the minimum duration of cold stimulus required for detecting abnormal *xdh‐1(ok3234)* mutant cold tolerance, we conducted a time course assay for cold tolerance (Appendix Fig S4G) and found that approximately half of *xdh‐1* mutants died upon cold stimulus lasting 1 h. This suggests that changes to cold tolerance can occur within a 1‐h period in *xdh‐1* mutant.

### XDH‐1 regulates normal cold tolerance in both AIN and AVJ

**Figure 2:**
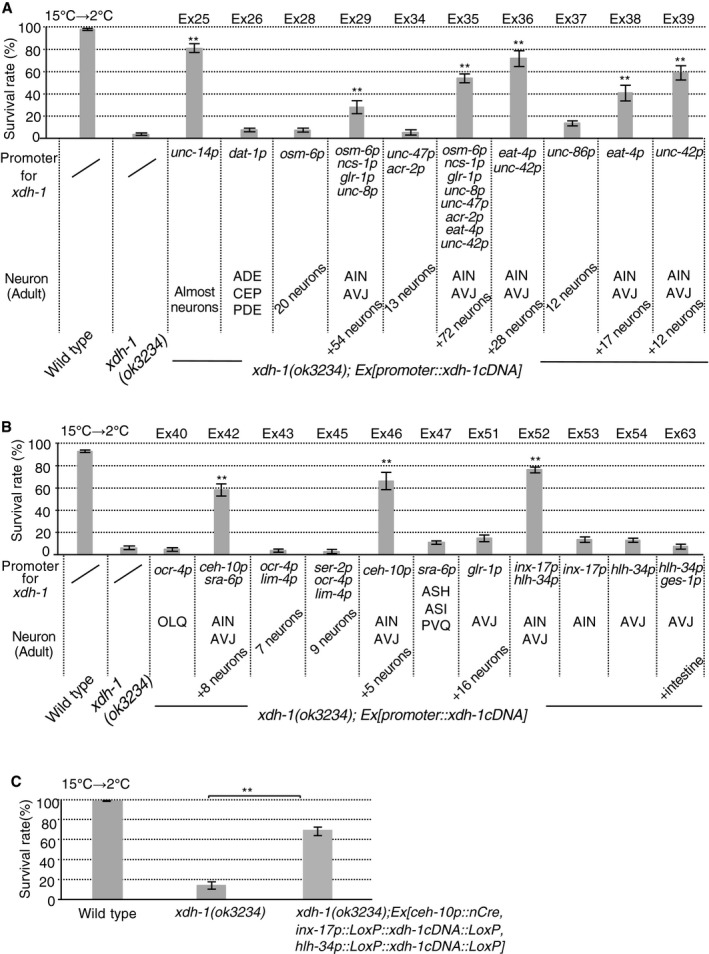
Cell‐specific rescue of abnormal *xdh‐1* mutant cold tolerance A, BExpression of *xdh‐1 cDNA* driven by various promoters. Irregular cold tolerance in *xdh‐1* was rescued by expressing *xdh‐1 cDNA* in both AVJ and AIN interneurons (number of assays ≥ 6). A part of data from wild type and *xdh‐1; Ex[unc‐14p::xdh‐1 cDNA]* are the same as those in Fig 1J, given that the experiments were conducted simultaneously.CExpression of *xdh‐1 cDNA* in only AIN and AVJ by Cre/LoxP recombination system (number of assays ≥ 9).Data information: In (A–C), the error bars indicate SEM. **P < 0.01 (Tukey–Kramer).Source data are available online for this figure.

To determine the neuron type required for *xdh‐1*‐dependent cold tolerance, we performed a series of cell‐specific rescue experiments by expressing *xdh‐1 cDNA* driven by various promoters (Fig 2 and Appendix Tables S2 and S3). Expressing *xdh‐1 cDNA* in almost all neurons driven by *unc‐14* promoter strongly rescued the abnormality of *xdh‐1* mutants (Fig 2A and Appendix Table S2: *xdh‐1; Ex25*). XDH‐1 expression in approximately 70 neuron pairs driven by eight types of promoter also rescued abnormal cold tolerance of the *xdh‐1* mutants (Fig 2A and Appendix Table S2: *xdh‐1; Ex35*). Likewise, expressing XDH‐1 in approximately 30 neuron pairs driven by *eat‐4* and *unc‐42* promoters achieved this rescue (Fig 2A and Appendix Table S2: *xdh‐1; Ex36*). Similar rescue phenomena of *xdh‐1* mutants were observed by expressing XDH‐1 driven by *eat‐4* or *unc‐42* promoter (Fig 2A and Appendix Table S2: *xdh‐1; Ex38, 39*). Both *eat‐4* and *unc‐42* promoters allowed XDH‐1 expression in ASH, AIN, and AVJ neurons (Fig 2A and Appendix Table S2: *xdh‐1; Ex38, 39*). Furthermore, XDH‐1 expression in several pairs of neurons containing ASH, AIN, and AVJ restored the defect of *xdh‐1* mutants (Fig 2B and Appendix Table S3: *xdh‐1; Ex42, 46*), whereas XDH‐1 expression in three neurons containing ASH did not achieve this (Fig 2B and Appendix Table S3: *xdh‐1; Ex47*). We found that decreased cold tolerance of *xdh‐1* mutants was rescued by the specific expression of XDH‐1 in both AIN and AVJ interneurons driven by *inx‐17* and *hlh‐34* promoters (Fig 2B and Appendix Table S3: *xdh‐1; Ex52,* and Appendix Fig S5A and B). Similarly, we found that decreased cold tolerance of *xdh‐1* mutants was rescued in the *xdh‐1* transgenic strain containing *xdh‐1 cDNA* only in AIN and AVJ by simultaneously expressing Cre sequence and loxP‐flanked stop with a promoter in combination (Fig 2C; *xdh‐1; Ex[ceh‐10p::nCre, inx‐17p::LoxP::xdh‐1 cDNA::LoxP, hlh‐34p::LoxP::xdh‐1 cDNA::LoxP]*). However, the expression of XDH‐1 in either AIN or AVJ alone did not achieve this rescue (Fig 2B and Appendix Table S3: *xdh‐1; Ex53, 54*). These results suggest that the function of XDH‐1 in both AIN and AVJ is required for normal cold tolerance.

The roles played by the AIN and AVJ interneuronal circuits remain unknown for many *C. elegans* behaviors. To explore possible involvement of AIN and AVJ functions in a behavior, chemotaxis toward AWA‐ or AWC‐sensed odorants was evaluated in *xdh‐1(ok3234)* mutants. Mutation of the *xdh‐1* gene was found to have no influence on attraction toward AWA‐sensed diacetyl or AWC‐sensed benzaldehyde (Appendix Fig S5C and D).

### Mechanoreceptor mutants exhibit abnormal cold tolerance

**Figure 3:**
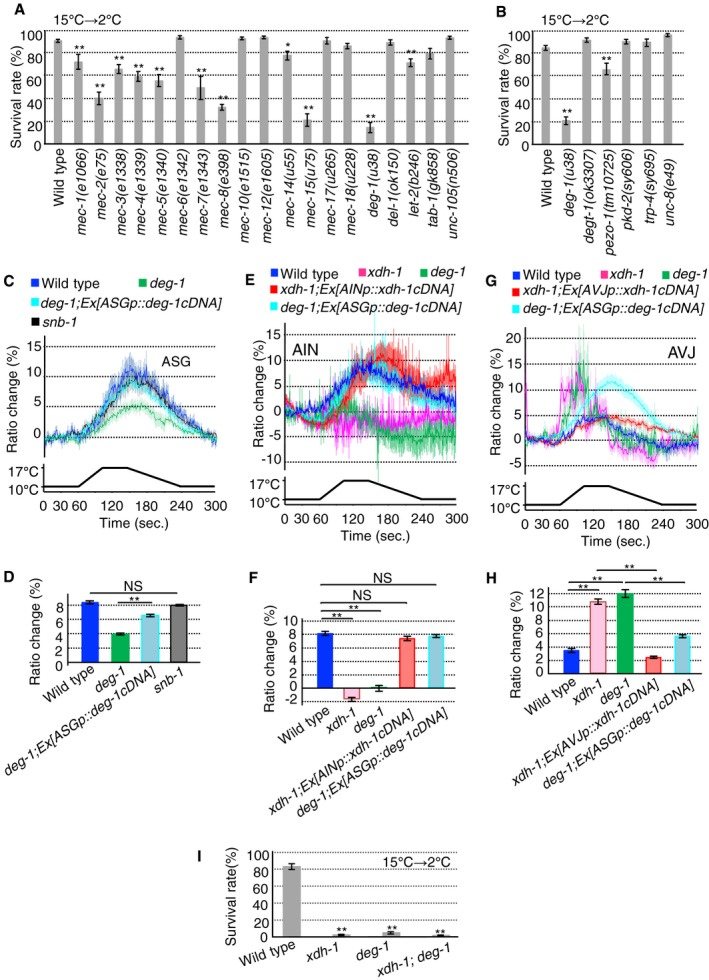
AIN and AVJ interneurons are involved in warm sensitivity A, B
*deg‐1* exhibited pronounced abnormality (number of assays ≥ 10). A part of data from the wild type are the same in (A and B), given that the experiments were conducted simultaneously.C, DASG Ca^2+^ imaging. (C) Average response to temperature stimulus. (D) Average change in cyan‐yellow ratio from 180 to 191 s in panel (C) (*n* ≥ 14 worms for each group). Color key is the same as that of the corresponding response curve in panel (C). As previously reported, wild‐type *deg‐1* gene restored the *u38* abnormal touch sensitivity, although *u38* is a dominant‐negative mutation 49.E, FAIN Ca^2+^ imaging. (E) Average response to temperature stimulus. (F) Average change in cyan‐yellow ratio from 120 to 131 s in panel (E) (*n* ≥ 14 worms for each group). Color key is the same as that of the corresponding response curve in panel (E).G, HAVJ Ca^2+^ imaging. (G) Average response to temperature stimulus. (H) Average change in cyan‐yellow ratio from 90 to 101 s in panel (G) (*n* ≥ 16 worms for each group). Color key is the same as that of the corresponding response curve in panel (G).ICold tolerance of *deg‐1; xdh‐1* double mutants. (number of assays ≥ 12).Data information: In (A–I), the error bars indicate SEM. (C–H) Ca^2+^ imaging was performed using yellow cameleon 3.60. (A) *P < 0.05; **P < 0.01 (Dunnett's test). (B, D, F, H, I) **P < 0.01 (Tukey–Kramer).Source data are available online for this figure.

Because AIN and AVJ are interneurons that receive a variety of sensory information, we hypothesized that temperature sensation by any upstream sensory neuron may affect the activity of AIN and/or AVJ. There are nine such neurons, five of which are known to be mechanoreceptor‐expressing sensory neurons. To determine whether these mechanoreceptor neurons are involved in cold tolerance, we tested the cold tolerance of mutants defective in various aspects of mechano‐transduction. Experimental evidence showed that mutation to any of a number of mechanoreceptor components could lead to abnormal cold tolerance (Fig 3A and B). Mutation to *deg‐1*, which encodes a trimeric degenerin/epithelial Na^+^ channel (DEG/ENaC)‐type mechanoreceptor, resulted in particularly severe cold tolerance dysfunction (Fig 3A and B).

We also conducted cold tolerance experiments using other types of mechanoreceptor mutants such as PIEZO encoded by *pezo‐1*, TRP channel encoded by *pkd‐2* and *trp‐4*, and DEG/ENaC encoded by *degt‐1* and *unc‐8*. *trp‐4* and *unc‐8* mutants displayed almost normal cold tolerance, while *pezo‐1* mutants showed a slightly abnormal one (Fig 3B). In addition, strongly abnormal cold tolerance was observed in the mutant lacking the *mec‐15* gene, which encodes an ortholog of human FBXW9 (F‐box and WD repeat domain containing 9) (Fig 3A); however, this protein is not a mechanoreceptor 37. We focused on a mechanoreceptor protein encoded by the *deg‐1* gene because its mutants exhibited the most remarkably abnormal cold tolerance phenotype (Fig 3A and B).

### DEG‐1 in ASG affects AIN and AVJ circuit in temperature signaling of cold tolerance

ASG is the sole sensory neuron pair upstream of AIN/AVJ that expresses DEG‐1, and is located in the head of *C. elegans*. Ca^2+^ imaging revealed that wild‐type ASG responds to temperature changes by increasing intracellular Ca^2+^ concentration (Fig 3C and D, Appendix Fig S6A and B). The temperature response of ASG was normal in a mutant with impairment of SNB‐1/synaptobrevin (Fig 3C and D), indicating that the responsiveness of ASG to warm temperatures probably does not require input from other neurons. In contrast, such a thermal response in *deg‐1* mutants was lower than in the wild type, which was rescued by the ASG‐specific expression of *deg‐1 cDNA* (Fig 3C and D, Appendix Fig S6A and B). These results suggest that DEG‐1 is involved in the response of ASG sensory neurons to temperature change.

Because DEG‐1 is expressed in some neurons besides ASG, such as AVG and PVC, we measured the neuronal responses of AVG and PVC neurons under temperature stimuli. We constructed a transgenic strain, *wild type; Ex[nmr‐1p::yc3.60]*, and measured the changes in Ca^2+^ concentration upon the application of temperature stimuli. Ca^2+^ concentrations in the AVG and PVC neurons were changed by temperature stimuli, suggesting that these neurons respond to temperature changes as well as ASG neurons (Appendix Fig S6C).

To investigate whether defective temperature sensation of ASG in *deg‐1* mutants causes abnormal neuronal activity in its downstream interneurons AIN and AVJ, we performed Ca^2+^ imaging using the cameleon as Ca^2+^ indicator. The AIN and AVJ Ca^2+^ concentrations in *deg‐1* mutants varied abnormally upon the application of a thermal stimulus compared with those in the wild type (Fig 3E–H). In *deg‐1* mutants, AIN activity diminished, while AVJ activity increased (Fig 3E–H). Moreover, the responsiveness of *xdh‐1* mutants AIN and AVJ was remarkably similar to the abnormal neural activities of these neurons in *deg‐1* mutants, and AIN and AVJ abnormalities in *xdh‐1* mutants were rescued by expressing XDH‐1 in AIN and AVJ, respectively (Fig 3E–H). We found that DEG‐1 expression in ASG of the *deg‐1* mutants restored the abnormally decreased AIN activity and also restored the abnormal rapidly elevated activation of AVJ, although a higher Ca^2+^ level was sustained after warming (Fig 3E–H). This suggested that DEG‐1's functions in ASG altered the temperature responses in AIN and AVJ, although it is possible that more complex neural signaling underlies the circuit calculation. These Ca^2+^ imaging results are also consistent with the finding of genetic epistasis between *xdh‐1* and *deg‐1* mutations that *deg‐1; xdh‐1* double mutants exhibited phenotypes comparable to those in mutants with either single mutation alone (Fig 3I). This in turn suggests that *xdh‐1* and *deg‐1* act within the same pathway.

### *xdh‐1* mutation genetically parallels to known cold tolerance mutations

XDH‐1 is active in the AIN and AVJ interneurons required for normal cold tolerance. We demonstrated genetic epistasis between the *xdh‐1* mutation and known cold tolerance mutations related to temperature signaling, to analyze the relationship between neural cells known to be involved in cold tolerance and the AIN and AVJ interneurons (Appendix Fig S6D). ASJ sensory neurons detect temperature and negatively regulate cold tolerance through insulin and hormonal signaling 5, 7. During ASJ thermosensation, the cyclic‐GMP‐gated channel encoded by *tax‐4* acts as a primary channel 5. *tax‐4* mutants cultivated at 20°C exhibited abnormally enhanced cold tolerance, but normal function was recovered upon expressing *tax‐4 cDNA* in the ASJ 5. We constructed *tax‐4; xdh‐1* double mutants. After cultivation at 15°C, the wild type and *tax‐4* mutants remained alive under cold stimulus, while *xdh‐1* mutants died under the same conditions (Appendix Fig S6D). *tax‐4; xdh‐1* double mutants exhibited an intermediate phenotype suggesting that *xdh‐1* mutation is genetically parallel to *tax‐4* mutation. These results are consistent with the downstream position of AIN and AVJ interneurons.

We also constructed *daf‐2; xdh‐1* double mutants. DAF‐2 is the sole insulin receptor in *C. elegans* and is active in both intestine and neurons for regulating cold tolerance. The wild type and *daf‐2* mutants cultivated at 15°C could survive at 2°C, but *xdh‐1* mutants could not. In *daf‐2; xdh‐1* double mutants, the *xdh‐1* mutation strongly suppressed *daf‐2* mutation (Appendix Fig S6D), suggesting that *xdh‐1* mutation is epistatic to *daf‐2*.

### Expressing DEG‐1 confers warm responsiveness to gustatory neuron and *Xenopus* oocyte

**Figure 4:**
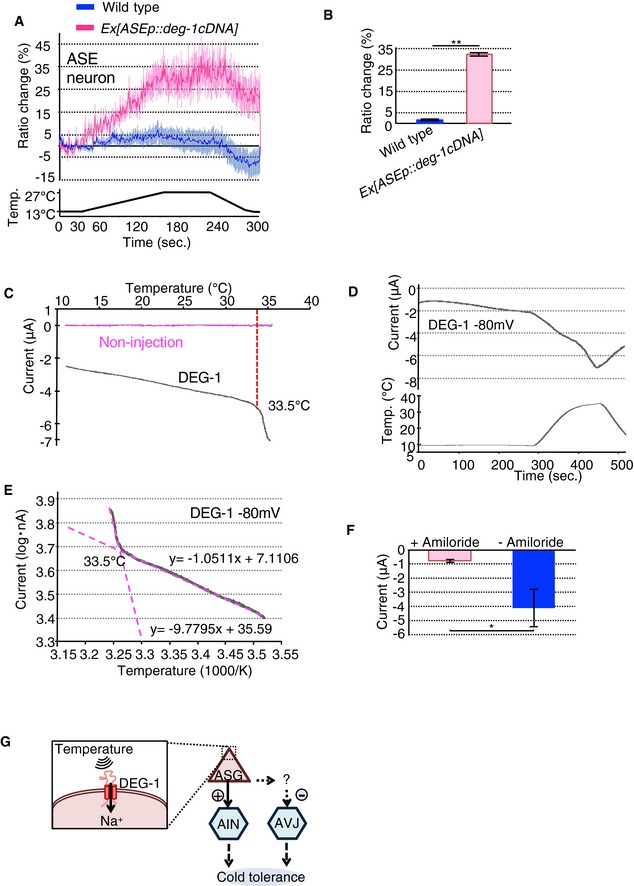
DEG‐1 is involved in cold tolerance and warm sensitivity A, BCa^2+^ imaging of non‐warm‐sensitive ASE neurons in wild type ectopically expressing DEG‐1 in ASE. (A) Average response to temperature stimulus. (B) Average change in cyan‐yellow ratio from 230 to 241 s in panel (A) (*n* ≥ 19 worms for each group).C–FReactions (representative current traces) to thermal stimulus in *Xenopus* oocytes expressing DEG‐1. (C) Heating phase relationship between current and temperature shown in panel (D). Data from noninjected oocytes (*n* = 8 oocytes). (D) Representative current (upper) and temperature (lower) traces (*n* = 8 oocytes). (E) Arrhenius plots from data in panel (D). Temperature threshold was determined by the intersection of the two extended lines shown in magenta (*n* = 8 oocytes). (F) Suppression of heat‐evoked currents by incubation in bath solution with amiloride, an inhibitor of DEG/ENaC ion channel (*n* ≥ 5 oocytes for each group).GA model for the neural circuit for cold tolerance modulated from ASG to AIN and AVJ neurons. ASG senses ambient temperature via DEG‐1, and directly and indirectly connects to AIN (Arrow) and AVJ (dotted‐line arrow), where ASG positively and negatively controls AIN and AVJ activities, respectively, which positively regulates cold tolerance.Data information: In (A, B, F), the error bars indicate SEM. (B, F) *P < 0.05; **P < 0.01 [*t*‐test (Welch)]. (A) Ca^2+^ imaging was performed using GCaMP8 with tag‐RFP.Source data are available online for this figure.

To determine whether mechanoreceptor DEG‐1 is involved in temperature sensation, we ectopically expressed DEG‐1 in the non‐warm‐sensitive ASE gustatory neuron and then measured the resulting intracellular Ca^2+^ dynamics. ASE gustatory neurons ectopically expressing DEG‐1 strongly responded to warming, while wild type ASE did not (Fig 4A and B). These results suggest that the ectopic expression of DEG‐1 is sufficient to confer warm responsiveness to the ASE gustatory neuron.

Next, we conducted electrophysiological experiments to determine the thermosensitivity of DEG‐1 by applying the two‐electrode voltage‐clamp recording method to *Xenopus* oocytes (Fig 4C–F). The DEG‐1 mechanoreceptor and its human homologue MDEG1 were expressed separately in *Xenopus* oocytes by injecting corresponding *cRNAs* for *deg‐1 cDNA* and *MDEG1 cDNA*. Thermal stimulus evoked internally directed current in these oocytes (Fig 4C–E, Appendix Fig S7A–C). Conversely, oocytes lacking DEG‐1 and MDEG1 did not show such currents (Fig 4C and Appendix Fig S7A, no injection) and an inhibitor of DEG/ENaC ion channel, amiloride, inhibited heat‐evoked currents of DEG‐1 (Fig 4F and Appendix Fig S8A–C). These results suggest that DEG‐1 and MDEG1 act as warm‐sensitive channels [Fig 4E and Appendix Fig S7C; average of temperature threshold: 32.0 ± 0.8°C for DEG‐1 (*n* = 8) and 31.0 ± 0.3°C for MDEG (*n* = 8)].

## Discussion

The elucidation of temperature tolerance mechanism of animals is an important for understanding an adaptive mechanism to tolerate external environments. The results in this study show that the XDH encoded by *xdh‐1* gene acts as a positive regulator of cold tolerance in *C. elegans*. Opposed functions of XDH‐1 in AIN and AVJ interneurons are a central in neural circuit calculation of cold tolerance. ASG sensory neuron is a functionally upstream interneuron of AIN and AVJ, and mechanoreceptor DEG‐1 in ASG is critical for temperature response of ASG. Ectopic expression of DEG‐1 in a gustatory neuron and *Xenopus* oocyte resulted in acquisition of warm sensitivity. Thus, the mechanoreceptor acts as an ambient temperature sensor in a neural circuit underlying the cold tolerance in *C. elegans* (Fig 4G).

### Opposite temperature‐dependent Ca^2+^ responses in AIN and AVJ of *xdh‐1* mutant

In this study, XDH‐1 expressed in both AIN and AVJ is required for normal cold tolerance, and AIN and AVJ interneurons exhibit mutually opposing activity under temperature stimulus (Fig 3E–H). This dual antagonism is proposed to be a central mechanism of *C. elegans* cold tolerance. However, why loss of XDH‐1 results in opposing changes in temperature‐dependent Ca^2+^ responses in the AIN and AVJ interneurons is unclear, then we discuss some possibilities regarding them. Although ROS concentration in the whole body in *xdh‐1* mutants was almost normal in this study (Appendix Fig S3A) and we could not measure ROS concentration at the single‐cell level, it is possible that decreased ROS or increased ROS in individual neurons could occur in *xdh‐1* mutants for the following reason. XDH acts enzymatically in the purine base salvage pathway and induces oxidative stress in the process of uric acid production 29, 38. However, uric acid functions as a potent antioxidant and protects against oxidative damage 39. These findings suggest that the loss of XDH causes decreased ROS directly or increased ROS indirectly. A previous report indicated that increased ROS enhances neuronal excitability cell‐autonomously 40. Based on these previous studies, we speculated that loss of XDH‐1 could cause decreased ROS directly or increased ROS indirectly, and endogenous molecular components of each neuron may determine what happens to ROS levels in respective neurons, leading to neural activity being inhibited or activated, although we could not measure ROS concentration at the single‐cell level.

### A model for the neural circuit for cold tolerance modulated by ASG, AIN, and AVJ neurons

The analysis in this study described that the neural circuit from ASG to AIN and AVJ neurons regulates temperature signaling in cold tolerance, in which XDH‐1‐ and DEG‐1‐mediated pathway is essential and this pathway is parallel to known ASJ‐mediated cold tolerance pathway containing TAX‐4 (Appendix Fig S6D). The ASG neuron partially responded to temperature changes in the *deg‐1* mutant (Fig 3C and D, Appendix Fig S6A and B). We considered that another temperature receptor(s) may be expressed in ASG neurons, and DEG‐1‐independent temperature signaling probably acts in ASG of the *deg‐1* mutant. AIN and AVJ interneurons, downstream of ASG, showed abnormal decrease and increase in their neuronal activities in the *deg‐1* mutant, respectively (Fig 3E–H). These abnormalities of AIN and AVJ in the *deg‐1* mutant were quite severe, similar to the abnormalities in the *xdh‐1* mutant (Fig 3E–H), even though ASG temperature responsiveness partially decreased in the *deg‐1* mutant (Fig 3C and D, Appendix Fig S6A and B). These abnormal AIN and AVJ thermal responses in the *deg‐1* mutant were rescued in the *deg‐1* mutant expressing wild type DEG‐1 in ASG (Fig 3E–H). Overall, partial abnormality of ASG in the *deg‐1* mutant induces severe abnormalities of AIN and AVJ, which may be sufficient to cause the drastic decrease in the survival rate associated with cold tolerance of the *deg‐1* mutant, a phenomenon similar to that of the *xdh‐1* mutant.

### Discussion of other temperature receptor for cold tolerance

The results in this study showed that mechanoreceptor DEG‐1 is sufficient to confer temperature responsiveness when ectopically expressed in non‐warm‐sensitive ASE gustatory neuron and *Xenopus* oocyte. We used *gcy‐5* promoter as a ASE promoter that induces gene expression in ASE right (ASER) specifically, which is located at right side of the head. Gong *et al* 41 recently reported that ASER neuron acts as a cold‐sensing neuron, in which a kainate‐type glutamate receptor, GLR‐3, functions as a cold receptor. We then discuss about possibilities in a role of ASER and GLR‐3 in cold tolerance. *che‐1* gene encodes a C2H2‐type zinc finger transcription factor orthologous to *Drosophila* GLASS essential for photoreceptor cell differentiation, and CHE‐1 is required for determining the identity and function of the ASER and ASEL neurons 42. Previous paper described that *che‐1* mutant defective in development of ASER and ASEL neurons showed normal cold tolerance 5. This suggests that ASER is not probably required for cold tolerance. Although we could not measure cold tolerance of *glr‐3* mutant, we previously reported the analysis of GOA‐1 5, 43, a trimeric G protein alfa subunit, which is downstream molecule of GLR‐3 in cold‐sensing signaling of ASER 41. *goa‐1* mutant showed abnormal cold tolerance, and its abnormality was rescued by expressing wild‐type *goa‐1 cDNA* in ASJ sensory neurons in *goa‐1* mutant, in which GOA‐1 signaling downstream of GLR‐3 in ASER is remaining defective 43. These indicate that GOA‐1‐mediated GLR‐3 signaling in ASER is not probably required for cold tolerance. Gong *et al* 41 described that ASER is a cold‐sensing neuron and is not responsive to warming stimuli. In the results of this our study, ectopic expression of DEG‐1 in ASER obtained warm sensitivity, suggesting that DEG‐1 is sufficient to confer warm responsiveness to the ASER neuron. These results are consistent with the results of electrophysiological analysis with *Xenopus* oocytes expressing DEG‐1 and its human homologue MDEG1 that both of them are capable to confer warm responsiveness. These results suggested that DEG‐1 confers warm‐sensing ability. Besides, other genetic and Ca^2+^ imaging studies in this study suggested that the function of DEG‐1 is involved in warm sensation of ASG, which affects downstream AIN and AVJ interneurons essential for cold tolerance.

### DEG‐1 is involved in ambient temperature sensation

Recording electrophysiological activity using two‐electrode voltage‐clamp method described thermal stimulus generated a Na^+^ current in *Xenopus* oocytes expressing DEG‐1 and its human homologue MDEG (Fig 4C–E, Appendix Fig S7A–C). Average of temperature threshold of 32°C was determined by the intersection of the two magenta lines per phases (Fig 4E), resulting that DEG‐1 is a protein capable of detecting temperature. It should be noted that the living temperature of *C. elegans* is between 13 and 27°C, while the *Xenopus* oocyte DEG‐1 reactions took place at 32°C. These discrepant responses may have been caused by the difference in membrane lipids between *C. elegans* and *Xenopus* or the intracellular/extracellular environment in electrophysiological measurement. Although we observed cold tolerance, the cold‐tolerant status of *C. elegans* is established during ambient temperature such as at 15°C or 25°C, and is not established at cold conditions, as previously reported 5. The above findings are thus consistent with the result in this study that DEG‐1 plays a role in sensing ambient temperature and in a non‐cold sensor.

Overall, the experiments performed in this study suggest that the DEG/ENaC‐type mechanoreceptor DEG‐1 acts as an ambient temperature sensor in ASG sensory neurons, which regulates AIN and AVJ interneurons to accomplish cold tolerance (Fig 4G). Many molecular systems are conserved throughout *C. elegans* to humans in evolution. The DEG/ENaC‐mediated temperature tolerance found in this study may provide a new insight for understanding body temperature response in human and other animals.

## Materials and Methods

### Strains

We used the following *C. elegans* strains: N2 Bristol England (as wild type) in all experiments, KHR066/RB2575 *flp‐17(ok3587) xdh‐1(chr1)*, KHR067/RB2379 *xdh‐1/F55B11.1(ok3234)*, VC883 *tag‐273(gk371)*, FX07280 *tbc‐9(tm7280)*, KHR069 *xdh‐1(chr1)*, CB1066 *mec‐1(e1066)*, CB75 *mec‐2(e75)*, CB1338 *mec‐3(e1338)*, CB1339 *mec‐4(e1339)*, CB1340 *mec‐5(e1340)*, CB1472 *mec‐6(e1342)*, CB2477 *mec‐7(e1343)*, CB398 *mec‐8(e398)*, CB1515 *mec‐10(e1515)*, CB3284 *mec‐12(e1605)*, TU55 *mec‐14(u55)*, TU75 *mec‐15(u75)*, TU265 *mec‐17(u265)*, TU228 *mec‐18(u228)*, TU38 *deg‐1(u38)*, NC279 *del‐1(ok150)*, DH246 *let‐2(b246)*, VC1812 t*ab‐1(gk858)*, MT1098 *unc‐105(n506)*, VC2633 *degt‐1(ok3307)*, FX010725 *pezo‐1(tm10725)*, PT8 *pkd‐2(sy606); him‐5(e1490)*, TQ296 *trp‐4(sy695)*, and CB49 *unc‐8(e49)*. See Appendix for further details.

### Statistical analysis

Cold tolerance testing was conducted on six or more plates for 3 or more nonconsecutive days. All error bars in the figures indicate standard error of the mean (SEM). All statistical analyses assumed a normal distribution and were performed using parametric tests, the Tukey–Kramer method, Dunnett's test, or the unpaired *t*‐test (Welch). Multiple comparisons were performed using one‐way ANOVA tested with the Tukey–Kramer method and Dunnett's test. Dunnett's test was performed to compare the groups represented by the leftmost bar in the graphs with the other groups. Comparisons between other pairs of groups were performed using the unpaired *t*‐test (Welch) (*P < 0.05; **P < 0.01).

### Cold tolerance assay

The cold tolerance assay was performed in accordance with previous reports 5, 7, 8, 44. For this assay, we placed well‐fed adult worms onto nematode growth medium (NGM) with 2% (w/v) agar, and then seeded the medium with *Escherichia coli* OP50 once the worms began laying eggs. Adults were removed after 16–24 h at 15°C, and progeny were left to mature for 120–130 h at 15°C. Before the next generation had hatched, plates containing fresh adult worms were counted after being placed on ice for 20 min, followed by transfer to a 2°C refrigerated cabinet (CRB‐41A; Hitachi, Tokyo, Japan) for 8–96 h. The temperature inside this cabinet was monitored using both digital and mercury thermometers. After cold stimulus, plates were either repeatedly transferred to a room‐temperature environment (total time of over 3 h) or stored at 15°C overnight. We then counted living and dead worms to calculate survival rates.

### Volatile odorant chemotaxis assay

Chemotaxis to volatile odorants was assayed in accordance with previous reports 45.

### Fatty acid composition

Lipids were extracted from synchronized cultures of adult worms and then transmethylated as described in previous studies 5, 9. Fatty acid methyl esters were analyzed by gas–liquid chromatography and identified by comparing peak retention times with authentic standards. Fatty acid compositions are presented as percentages by weight.

### Confocal microscopy analysis

The following procedure was used to prepare samples for confocal microscopy: 2% (w/v) agarose gel on a glass micro‐slide was covered with 10 μl of 100 mM NaN_3_. A few adult or larval worms were then placed on the gel. The gel was covered by glass, and fluorescent images were analyzed by confocal laser microscopy (FV1000‐IX81 with GaAsP PMT; Olympus, Japan), using FV10‐ASW software (Olympus, Japan).

### Germline transformation

Germline transformations were conducted as described previously 46, with co‐injection mixtures consisting of experimental DNA at various concentrations (5–100 ng/μl) and pRF04 *rol‐6gf*, pAK62 *AIYp::gfp*, or pKDK66 *ges‐1p::nls::gfp* as a transgenic marker at 30–50 ng/μl.

### ROS level measurement

Reactive oxygen species levels were quantified using the cell‐permeable, nonfluorescent probe 2′,7′‐dichlorofluorescein diacetate (H_2_DCF‐DA) (Thermo Fisher Scientific, USA). We used synchronized, counted adult worms cultivated at 15°C. Approximately 200–250 worms were harvested and washed in M9 buffer. Bacteria (OP50) were removed by three repeated washes, after which the resulting fluid was centrifuged at low speed. Animals were homogenized in 400 μl of PBS with 0.1% Tween 20 on ice using a glass homogenizer. To measure fluorescence, we used a fluorescence microplate reader (CFX96 Real‐Time System and C1000 Thermal cycler; Bio‐Rad, USA) every 10 min for 1 h with an excitation wavelength of 485 nm and an emission wavelength of 535 nm at 15°C.

### Molecular biology

pNTN020 *xdh‐1p::xdh‐1 genomic gene::gfp* contains the *xdh‐1* full‐length gene and a 3,346 bp segment upstream of it amplified from the wild‐type genome by PCR. GFP was inserted into the *xdh‐1* full‐length gene, excluding the stop codon. pNTN026 *xdh‐1p::gfp* contains the 3,346 bp upstream promoter sequence and the 3′‐UTR of *xdh‐1* amplified by PCR from pNTN020. GFP was then inserted by pPDF95.75. pNTN032 *pgp‐12p::dsRedm* contains a 3,500 bp upstream promoter sequence for the *pgp‐12* gene as well as dsRedm. pNTN058 *xdh‐1p::xdh‐1 cDNA::gfp* contains a 3,346 bp upstream promoter sequence for the *xdh‐1* gene and the *xdh‐1 cDNA*. *xdh‐1 cDNA* stop codon was replaced by GFP via pNTN036. *xdh‐1* PCR 1, 2, and 3 contain 1,772, 952, and 428 bp *xdh‐1* upstream promoter sequences, respectively. *xdh‐1 cDNA::gfp* was amplified by PCR from pNTN058, which was used for the transgenic rescue experiment. pNTN118 *xdh‐1p::dsRedm* contains the *xdh‐1* promoter and dsRedm. pNTN027 contains a Kozak sequence, the *xdh‐1 cDNA* that was amplified by PCR from the cDNA library, and the 3′‐UTR of the *unc‐54* gene. The promoter sequences, *unc‐14p* (1.4 kb), *pgp‐12p* (3.5 kb), *ges‐1p* (3.3 kb), *xdh‐1p* (3.4 kb), *dat‐1p* (0.7 kb), *osm‐6p* (2 kb), *ncs‐1p* (3.1 kb), *glr‐1p* (5.4 kb), *unc‐8p* (4.2 kb), *unc‐47p* (0.3 kb), *acr‐2p* (3.4 kb), *eat‐4p* (6.4 kb), *unc‐42p* (3 kb), *unc‐86p* (3.6 kb), *ocr‐4p* (4.8 kb), *ceh‐10p* (3.5 kb), *sra‐6p* (3.8 kb), *lim‐4p* (3.6 kb), *ser‐2p* (4.1 kb), *inx‐17p* (1.2 kb), and *hlh‐34p* (2.5 kb), were inserted upstream of pNTN027 *xdh‐1 cDNA*, to create pNTN034, 035, 036, 046, 047, 048, 049, 050, 051, 052, 053, 054, 055, 057, 059, 060, 061, 063, 064, 067, and 068 plasmids, respectively, for cellular experimentation. pNTN075 *hlh‐34p::yc3.60* contains the 2.5 kb *hlh‐34p* gene and the *yc3.60* gene. pNTN106 *gcy‐5p::deg‐1 cDNA* contains the *gcy‐5* promoter received from Dr. Yuichi Iino, the University of Tokyo. pNTN116 contains the *inx‐17* promoter, the *yc3.60* gene, and the 3′‐UTR of the *let‐858* gene. pNTN123 *gcy‐21p::yc3.60* contains the 1,403 bp upstream promoter sequence of the *gcy‐21* gene amplified by PCR from the wild‐type genome, which was created by replacing the *hlh‐34p* gene of pNTN075 with *gcy‐21p*. Previous reports described the *gcy‐21p::gfp* construct containing the first and second exons and the first intron as inducing the expression of GFP strongly in ASG and weakly in ADL. However, the *gcy‐21p::gfp* construct excluding all exons and introns induces GFP expression in ASG only. We therefore used *gcy‐21p* as an ASG‐specific promoter. pNTN126 *gcy‐21p::deg‐1 cDNA* contains the 1,403 bp upstream promoter sequence of *gcy‐21* and *deg‐1 cDNA*. pMIU34 *flp‐6p::CeG‐CaMP8* contains a 2,680 bp upstream promoter sequence for the *flp‐6* gene and G‐CaMP8 that is codon‐optimized for *C. elegans* (*CeG‐CaMP8*). *deg‐1 cDNA* was inserted into a pGEMHE vector containing *Xenopus* beta‐globin 5′‐ and 3′‐UTR for electrophysiological recording (pNTN119). *MDEG cDNA* was inserted into a pGEMHE vector containing *Xenopus* beta‐globin 5′ and 3′ UTR for electrophysiological recordings (pNTN125). pNTN143 *ceh‐10p::nCre* contains the *ceh‐10* promoter sequence and the *nCre* sequence. pNTN144 *hlh‐34p::LoxP::xdh‐1 cDNA::LoxP* contains the *hlh‐34* promoter sequence, *xdh‐1 cDNA*, sandwiched between two LoxP‐flanked stop sequences. pNTN145 *inx‐17p::LoxP::xdh‐1 cDNA::LoxP* contains the *inx‐17* promoter sequence, *xdh‐1 cDNA*, also sandwiched between two LoxP‐flanked stop sequences. pNTN159 *nmr‐1p::yc3.60* contains 5 kb of upstream *nmr‐1* gene sequence including the first five codons and *yc3.60*.

### Two‐electrode voltage‐clamp recording in *Xenopus* oocytes

*deg‐1 cRNA* and *MDEG cRNA* were separately injected into oocytes and incubated at 18°C for 3–6 days before electrophysiological recordings were performed. We held the membrane potential at −80 mV and recorded the macroscopic current using the two‐electrode voltage‐clamp technique with a bath clamp amplifier (OC‐725C; Warner Instruments, USA) and pClamp software (Molecular Devices, USA) in bath solution containing 100 mM NaCl, 2 mM MgCl_2_, and 10 mM HEPES (pH 7.3). Temperature stimulation was regulated using a lab‐made temperature controller with a range of 10–35°C and was monitored by both a thermistor probe adjacent to the oocytes and a thermometer (Digital Thermometer PTC‐401; Unique Medical, Japan). An Arrhenius plot was created, indicating the current amplitude induced by temperature changes on the *y*‐axis (log scale) versus the inverse of temperature on the *x*‐axis (1,000/K). Temperature thresholds were determined by the intersection of the two linear regions (magenta lines), and all thresholds were then averaged. As a negative control experiment, we used amiloride, an inhibitor of DEG/ENaC ion channel, and oocytes incubated at 18°C in bath solution with 500 μM amiloride (Sigma‐Aldrich) for 48 h.

### *In vivo* Ca^2+^ imaging

*In vivo* Ca^2+^ imaging was performed in accordance with previous reports 5, 8, 47. We used yellow cameleon (yc3.60) and GCaMP8 as genetically encodable Ca^2+^ indicators. When using GCaMP8, we co‐expressed tag‐RFP (pKOB006 *gcy‐5p::tag‐RFP*) to measure the fluorescence ratio between GCaMP and tag‐RFP 48. Adult worms cultivated at 15°C that express the Ca^2+^ indicator in one or more neurons were glued to a 2% (w/v) agar pad on glass, immersed in M9 buffer, and covered by a cover glass. Cyan (CFP) and yellow (YFP) fluorescence by YC3.60, and green (GCaMP8) and red (tag‐RFP) fluorescence were simultaneously captured using an EM‐CCD camera, EVOLVE512 (Photometrics, USA). Changes in intracellular Ca^2+^ concentration were measured as the yellow/cyan fluorescence ratio for YC3.60 or green/red fluorescence ratio for GCaMP8. See Appendix Supplementary Methods for more detail.

## Author contributions

NT, AO, KO, AKa, YM, AT, YF, and AKu performed the experiments; NT, AO, AT, YF, and AKu designed the experiments, interpreted the results, and wrote the final report.

## Conflict of interest

The authors declare that they have no conflict of interest.

## Supporting information

Appendix

Source Data for Appendix

Review Process File

Source Data for Figure 1

Source Data for Figure 2

Source Data for Figure 3

Source Data for Figure 4

## Data Availability

All data required to evaluate the study conclusions can be found in either the main text or the Appendix Supplementary Methods. Requests for further information should be addressed to A.O. or A. Kuhara. Information regarding data, figures, or other research findings may be obtained by contacting the corresponding authors. DNA sequencing data have been deposited in the DRA of DDBJ with accession number DRA002599 (http://trace.ddbj.nig.ac.jp/DRASearch/submission?acc=DRA002599).
