# Supplementary material for: The mechanoreceptor DEG‐1 regulates cold tolerance in Caenorhabditis elegans
Source: EMBO Rep. 2020 Feb 3;21(3):e48671. doi: 10.15252/embr.201948671 (PMC7054665; doi:10.15252/embr.201948671)
Supplement: Supplementary file 1 — Appendix [file EMBR-21-e48671-s001.pdf]

**Table of contents**

| | |
|---|---|
| **Appendix Supplementary Text** | **2–8** |
| **Appendix Figure S1** | **9–10** |
| **Appendix Figure S2** | **11–12** |
| **Appendix Figure S3** | **13–14** |
| **Appendix Figure S4** | **15–16** |
| **Appendix Figure S5** | **17–18** |
| **Appendix Figure S6** | **19–20** |
| **Appendix Figure S7** | **21** |
| **Appendix Figure S8** | **22** |
| **Appendix Table S1** | **23** |
| **Appendix Table S2** | **24** |
| **Appendix Table S3** | **25** |

## Appendix Supplementary Text

### Genetic mapping of key mutation in novel cold tolerance mutant *chr1*

We previously reported data from DNA microarray analysis for the purpose of isolating new molecules involved in cold tolerance [1]. The expression levels of multiple genes changed within 3 h of increasing the temperature from 15°C to 25°C [1] (GEO accession number: GSE81409). Measuring the cold tolerance phenotypes of 15 mutant strains, we found that RB2575 *flp-17(ok3587)* mutants exhibited nonfunctional cold tolerance (Appendix Fig S1A). Null mutant *flp-17(n4894)* did not exhibit abnormal cold tolerance (Appendix Fig S1B), and the abnormal cold tolerance of *flp-17(ok3587)* was not restored by the expression of genomic *flp-17* (Appendix Fig S1C). We therefore hypothesized that background mutation(s) other than *flp-17(ok3587)* in the RB2575 strain led to abnormal cold tolerance; we then proceeded to decode the entire RB2575 strain genome using next-generation sequencing (NGS) (accession number: DRA 002599). This sequencing revealed 652 single-nucleotide polymorphisms (SNPs), upon comparison with wild-type N2. Referencing these SNPs, mutations responsible for abnormal cold tolerance were mapped onto the right arm of chromosome IV (Appendix Fig S1D). To further narrow down the mapping region, we obtained additional recombinant lines #bc5-1, #bc5-2, and KHR066 (Appendix Fig S1E). Based on the cold tolerance phenotype and SNP patterns of these recombinants, responsible genes were mapped between 6.13 and 16.24 cM, a range that includes only five mutations among four genes (Appendix Figs S1E and F). By outcrossing N2 and KHR066, we removed

the *flp-17(ok3587)* mutation from KHR066 and named the resulting strain containing the five mutations among four genes KHR069 (Appendix Figs S1E and F). KHR069 animals still exhibited abnormal cold tolerance similar to that of RB2575 (Appendix Fig S1G); we named this cold tolerance mutation "*chr1*" (Appendix Fig S1G).

**Methods used for the work described in this Appendix Supplementary Text**

**Statistical analysis**

Cold tolerance tests were performed using eight plates or more and were carried out on 3 or more different days. All error bars in the figures indicate the standard error of the mean (SEM). All statistical analyses were performed using parametric tests, the Tukey–Kramer method, Dunnett's test, or the unpaired *t*-test (Welch), under an assumption of normally distributed data. Multiple comparisons were performed using one-way ANOVA with comparisons tested by the Tukey–Kramer method and Dunnett's test. Dunnett's test was performed to compare the groups represented by the left-most bar in the graphs with the other groups, while comparisons between other groups were performed using the unpaired *t*-test (Welch). *p < 0.05; **p < 0.01.

**Whole-genome DNA sequencing**

Whole-genome DNA sequencing was performed in accordance with previous reports [2]. Worms for genome extraction were cultured in NGM under well-fed conditions at 15°C. High-quality genomic DNA was extracted using a Gentra Puregene Tissue Kit (QIAGEN, Hilden, Germany), in accordance with the manufacturer's instructions. The

RB2575 and N2 genomic sequences were obtained using an Illumina Hiseq 2500 platform and a TruSeq DNA PCR-Free LT Sample Prep Kit (Illumina, San Diego, CA, USA), which generated 50–130 million reads (each read was 150 bp in length). Over 90% of the genome was sequenced at a coverage rate of 10×. The DNA sequence data reported in this paper have been deposited in the DNA Data Bank of Japan Sequence Read Archive (accession no. DRA 002599).

**Mapping gene responsible for *chr1* mutation**

We identified the gene responsible for the *chr1* mutation given the large phenotypic differences between wild-type animals and *chr1* mutants. Next, we created *wild-type; Ex[pKDK66 ges-1p::nls-gfp, pAK62 AIYp::GFP]* and crossed it with a *chr1* mutant. $F_1$ heterozygotes were placed on fresh NGM and incubated from the egg stage to L4/early adulthood. Animals expressing the GFP marker were isolated as $F_2$, yielding 20 recombinant lines. We then observed the phenotypes of these 20 recombinant lines from $F_3$ to $F_4$, finding that some exhibited abnormal cold tolerance. Finally, we crossed *wild-type; Ex[pKDK66 ges-1p::nls-gfp, pAK62 AIYp::GFP]* with one of the strains with abnormal cold tolerance. Recombinant lines, #bc5-1, #bc5-2, and KHR066, were constructed by repeating this procedure a total of five times.

We identified SNP patterns between the wild-type and the three recombinant strains by restriction-fragment-length polymorphism (RFLP) [3] or direct sequencing.

**Strains**

**Strains in Figs 1–4**

The *C. elegans* N2 (Bristol) strain was used as the wild-type in all experiments in this study. In addition, the following mutant strains were used: KHR066/RB2575 *flp-17(ok3587) xdh-1(chr1)*, KHR067/RB2379 *xdh-1/F55B11.1(ok3234)*, VC883 *tag-273(gk371)*, FX07280 *tbc-9(tm7280)*, KHR069 *xdh-1(chr1)*, CB1066 *mec-1(e1066)*, CB75 *mec-2(e75)*, CB1338 *mec-3(e1338)*, CB1339 *mec-4(e1339)*, CB1340 *mec-5(e1340)*, CB1472 *mec-6(e1342)*, CB2477 *mec-7(e1343)*, CB398 *mec-8(e398)*, CB1515 *mec-10(e1515)*, CB3284 *mec-12(e1605)*, TU55 *mec-14(u55)*, TU75 *mec-15(u75)*, TU265 *mec-17(u265)*, TU228 *mec-18(u228)*, TU38 *deg-1(u38)*, NC279 *del-1(ok150)*, DH246 *let-2(b246)*, VC1812 t*ab-1(gk858)*, MT1098 *unc-105(n506)*, VC2633 *degt-1(ok3307)*, FX010725 *pezo-1(tm10725)*, PT8 *pkd-2(sy606); him-5(e1490)*, TQ296 *trp-4(sy695)*, and CB49 *unc-8(e49)*, *xdh-1(ok3234); deg-1(u38)*, *N2; Ex [xdh-1p::gfp, pRF04], N2; Ex[hlh-34p::dsRedm, xdh-1p(1772bp)::xdh-1 cDNA::gfp], N2; Ex[xdh-1p::dsRedm, inx-17p::yc3.60::let-858UTR], xdh-1(chr1); Ex[irg-6p::irg-6 genomic gene, pAK62, pKDK66], xdh-1(ok3234); Ex[pRF04], xdh-1(chr1); Ex[xdh-1p::xdh-1 genomic gene::gfp, pRF04], xdh-1(ok3234);Ex [xdh-1p::xdh-1 genomic gene::gfp, pRF04], xdh-1(ok3234); Ex[pAK62, pKDK66], xdh-1(ok3234);Ex[unc-14p::xdh-1 cDNA, pAK62, pKDK66], xdh-1(ok3234); Ex[pgp-12p::xdh-1 cDNA, pAK62, pKDK66], xdh-1(ok3234); Ex[ges-1p::xdh-1 cDNA, pAK62, pKDK66], xdh-1(ok3234); Ex[xdh-1p::xdh-1 cDNA, pAK62, pKDK66], xdh-1(ok3234); Ex[dat-1p::xdh-1 cDNA, pAK62, pKDK66], xdh-1(ok3234); Ex[osm-6p::xdh-1 cDNA, pAK62, pKDK66], xdh-1(ok3234); Ex[osm-6p::xdh-1 cDNA,*

*ncs-1p::xdh-1 cDNA, glr-1p::xdh-1 cDNA, unc-8p::xdh-1 cDNA, pAK62, pKDK66], xdh-1(ok3234); Ex[unc-47p::xdh-1 cDNA, acr-2p::xdh-1 cDNA, pAK62, pKDK66], xdh-1(ok3234); Ex[osm-6p::xdh-1 cDNA, ncs-1p::xdh-1 cDNA, glr-1p::xdh-1 cDNA, unc-8p::xdh-1 cDNA, unc-47p::xdh-1 cDNA, acr-2p::xdh-1 cDNA, eat-4p::xdh-1 cDNA, unc-42p::xdh-1 cDNA, pAK62, pKDK66], xdh-1(ok3234); Ex[eat-4p::xdh-1 cDNA, unc-42p::xdh-1 cDNA, pAK62, pKDK66], xdh-1(ok3234); Ex[unc-86p::xdh-1 cDNA, pAK62, pKDK66], xdh-1(ok3234); Ex[eat-4p::xdh-1 cDNA, pAK62, pKDK66], xdh-1(ok3234); Ex[unc-42p::xdh-1 cDNA, pAK62, pKDK66], xdh-1(ok3234); Ex[ocr-4p::xdh-1 cDNA, pAK62, pKDK66], xdh-1(ok3234); Ex[ceh-10p::xdh-1 cDNA, sra-6p::xdh-1 cDNA, pAK62, pKDK66], xdh-1(ok3234); Ex[ocr-4p::xdh-1 cDNA, lim-4p::xdh-1 cDNA, pAK62, pKDK66], xdh-1(ok3234); Ex[ser-2p::xdh-1 cDNA, ocr-4p::xdh-1 cDNA, lim-4p::xdh-1 cDNA, pAK62, pKDK66], xdh-1(ok3234); Ex[ceh-10p::xdh-1 cDNA, pAK62, pKDK66], xdh-1(ok3234); Ex[sra-6p::xdh-1 cDNA, pAK62, pKDK66], xdh-1(ok3234); Ex[glr-1p::xdh-1 cDNA, pAK62, pKDK66], xdh-1(ok3234); Ex[inx-17p::xdh-1 cDNA, hlh-34p::xdh-1 cDNA, pAK62, pKDK66], xdh-1(ok3234); Ex[inx-17p::xdh-1 cDNA, pAK62, pKDK66], xdh-1(ok3234); Ex[hlh-34p::xdh-1 cDNA, pAK62, pKDK66], xdh-1(ok3234); Ex[hlh-34p::xdh-1 cDNA, ges-1p::xdh-1 cDNA, pAK62, pKDK66], xdh-1(ok3234); Ex [ceh-10p::nCre, inx-17p::LoxP::xdh-1 cDNA::LoxP, hlh-34p::LoxP::xdh-1 cDNA::LoxP], N2; Ex[hlh-34p::yc3.60, pRF04], xdh-1(ok3234); Ex[hlh-34p::yc3.60, pRF04], deg-1(u38); Ex[hlh-34p::yc3.60, pRF04], N2; Ex[inx-17p::yc3.60::let-858UTR], xdh-1(ok3234); Ex[inx-17p::yc3.60::let-858UTR], deg-1(u38); Ex[inx-17p::yc3.60::let-858UTR], N2;*

*Ex[gcy-21p::yc3.60], deg-1(u38); Ex[gcy-21p::yc3.60], deg-1(u38); Ex[gcy-21p::yc3.60, gcy-21p::deg-1 cDNA], N2; Ex[flp-6p::GCaMP8, gcy-5p::tagRFP], N2; Ex[flp-6p::GCaMP8, gcy-5p::tagRFP, gcy-5p::deg-1 cDNA].*

**Strains used for the work described in Appendix figs**

RB791 *hsp-16.48(ok577)*, FX05192 *F08A8.3/acox-3(tm5192)*, FX02994 *F36H1.5/hrg-4(tm2994)*, WM159 *T23D8.7(tm1163)*, FX03455 *K08F8.1/mak-1(tm3455)*, RB2207 *K08F8.1/mak-1(ok2987)*, VC3059 *ZK6.11(ok3738)*, FX04022 *sms-3(tm4022)*, RB2549 *sms-3(ok3540)*, FX07132 *B0222.9/gad-3(tm7132)*, FX03007 *C52B11.5(tm3007)*, VC1290 *nhr-125(gk578)*, FX01987 *F54F3.3/lipl-1(tm1987)*, FX06528 *C52D10.12/cnnm-1(tm6528)*, KHR066/RB2575 *flp-17(ok3587) xdh-1(chr1)*, MT15933 *flp-17(n4894)*, KHR069 *xdh-1(chr1)*, KHR067/RB2379 *xdh-1/F55B11.1(ok3234)*, CB1370 *daf-2(e1370)*, FX00776 *sod-1(tm776)*, FX03294 *gst-4(tm3294)*, CB767 *bli-3(e767)*, TK22 *mev-1(kn1)*, CX0010 *osm-9(ky10)*, FK127 *tax-4(p678), tax-4(P678); xdh-1(ok3234), daf-2(e1370); xdh-1(ok3234), flp-17(ok3587) xdh-1(chr1); Ex[pAK62, pKDK66], flp-17(ok3587) xdh-1(chr1); Ex[flp-17p::flp-17 genomic gene, pAK62, pKDK66], N2; Ex[xdh-1p::gfp], N2; Ex[xdh-1p::gfp, pgp-12p::dsRedm], xdh-1(ok3234); Ex[pRF04], xdh-1(ok3234); Ex[xdh-1p(3346bp)::xdh-1 cDNA::gfp, pRF04], xdh-1(ok3234); Ex[xdh-1p(1772bp)::xdh-1 cDNA::gfp, pRF04], xdh-1(ok3234); Ex[xdh-1p(952bp)::xdh-1 cDNA::gfp, pRF04], xdh-1(ok3234);*

*Ex[xdh-1p(428bp)::xdh-1 cDNA::gfp, pRF04], N2; Ex[hlh-34p::dsRedm, xdh-1p(1772bp)::xdh-1 cDNA::gfp], N2; Ex[nmr-1p::yc3.60, pRF04].*

**References for this Appendix Supplementary Text**

1. Sonoda S, Ohta A, Maruo A, Ujisawa T, Kuhara A (2016) Sperm Affects Head Sensory Neuron in Temperature Tolerance of Caenorhabditis elegans. *Cell Rep* **16**: 56-65

2. Okahata M, Ohta A, Mizutani H, Minakuchi Y, Toyoda A, Kuhara A (2016) Natural variations of cold tolerance and temperature acclimation in Caenorhabditis elegans. *J Comp Physiol B* **186**: 985-998

3. Wicks SR, Yeh RT, Gish WR, Waterston RH, Plasterk RH (2001) Rapid gene mapping in Caenorhabditis elegans using a high density polymorphism map. *Nat Genet* **28**: 160-4

## Appendix Supplementary Figures

# Appendix Figure S1

A

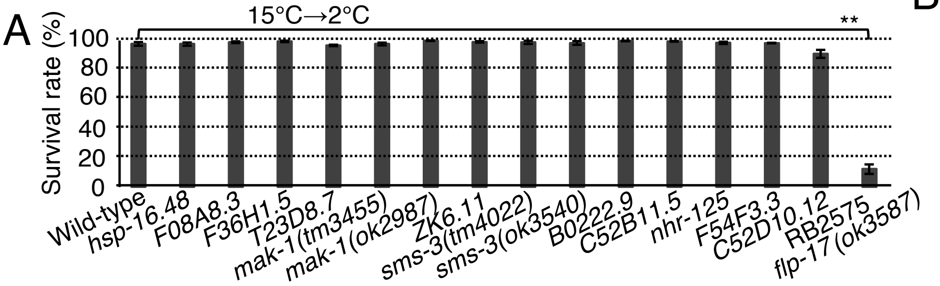


B

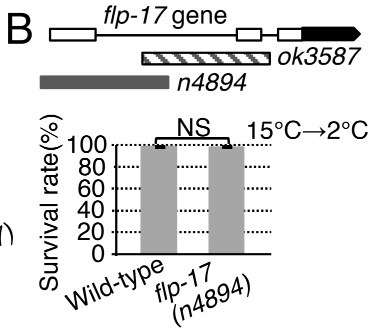


C

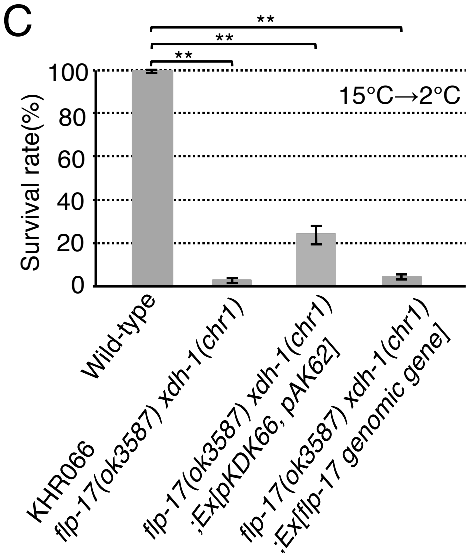


D

| | position | Abnormal phenotype strain genome pool |
|---|---|---|
| Chromosome I | 4377261 | Wild-type |
| | 6290450 | Wild-type/RB2575 |
| | 14874142 | Wild-type/RB2575 |
| Chromosome II | 4795820 | Wild-type |
| | 6948145 | Wild-type |
| | 1540306 | Wild-type/RB2575 |
| Chromosome III | 592022 | |
| | 4594988 | Wild-type/RB2575 |
| | 8883292 | Wild-type/RB2575 |
| Chromosome IV | 1981523 | Wild-type/RB2575 |
| | 7663908 | Wild-type/RB2575 |
| | 14412775 | RB2575 |
| Chromosome V | 2638723 | Wild-type/RB2575 |
| | 8773000 | Wild-type/RB2575 |
| | 17057384 | Wild-type/RB2575 |
| Chromosome VI | 2029668 | Wild-type/RB2575 |
| | 9299203 | |
| | 13054947 | Wild-type/RB2575 |

E

| | Genotype | | | | | | | | | |
|---|---|---|---|---|---|---|---|---|---|---|
| cM | –12.45 | 3.38 | 3.96 | 4.83 | 6.13 | 6.46 | 9.30 | 11.87 | 12.18 | 16.24 |
| Strain | | | | | | *irg-6* | *tbc-9* | *F55B1.1* | *tag-273* | *flp-17* |
| #bc5-1 | Wild-type | WT/RB | WT/RB | WT/RB | Unknown | Unknown | Unknown | RB2575 | RB2575 | RB2575 |
| #bc5-2 | Wild-type | RB2575 | RB2575 | RB2575 | RB2575 | RB2575 | RB2575 | RB2575 | RB2575 | RB2575 |
| KHR066 | Wild-type | Wild-type | Wild-type | Wild-type | Wild-type | Unknown | RB2575 | RB2575 | RB2575 | RB2575 |
| KHR069 | Wild-type | Wild-type | Wild-type | Wild-type | Wild-type | Unknown | RB2575 | RB2575 | Unknown | Wild-type |

F

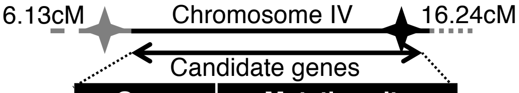


| Gene | Mutation site |
|---|---|
| *irg-6* | Splicing site |
| *tbc-9* | Amino acid change |
| *F55B11.1* | Splicing site & amino acid change |
| *tag-273* | Amino acid change |

G

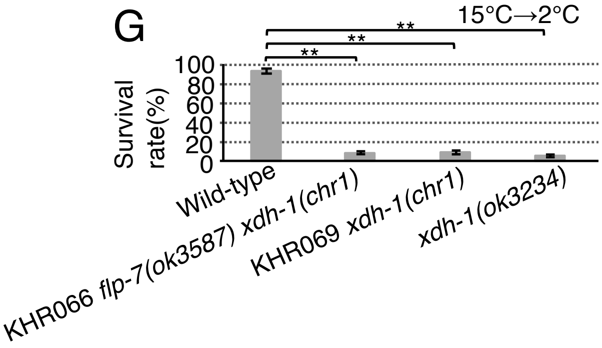

**Appendix Figure S1. Isolation of cold tolerance regulator xanthine dehydrogenase (XDH-1) via genetic mapping.**

(**A**) Cold tolerance was tested in 15 mutants that exhibited altered gene expression after a 3-h increase in temperature from 15°C to 25°C. RB2575 *flp-17(ok3587)* mutants demonstrated abnormal cold tolerance (number of assays ≥ 9).

(**B**) Upper: Schematic of the *flp-17* gene with exons (white box), 3′ UTR (black box), 0.7 kb *ok3587* deletion (stripe box), and 0.9 kb n4894 deletion (gray box). Lower: *flp-17(n4894)* mutants exhibiting abnormal cold tolerance (number of assays ≥ 9).

(**C**) Transgenic rescue of *flp-17* mutants with wild-type *flp-17* genomic fragment containing native promoter sequence **(**number of assays ≥ 9).

(**D**, **E**) In the genotype columns, "Wild-type" and "RB2575" indicate wild-type homozygote genotype and RB2575-type SNPs, respectively, whereas "Wild-type/RB2575" indicates the wild-type/RB2575 heterozygote at that position. Blank fields indicate undefined genotypes obtained by snip-SNP methods.

(**D**) Rough mapping of KHR066 on chromosomes I–V and X.

(**E**) Genotypes of each recombinant are indicated as cM on chromosome IV.

(**F**) Mapping region of KHR069. Four genes containing polymorphisms on the ORF or splicing site were located between 6.13 and 16.24 cM in chromosome IV.

(**G**) Cold tolerance was tested in two *xdh-1* mutant alleles. *xdh-1(chr1)* and *xdh-1(ok3234)* mutants exhibited abnormal cold tolerance (number of assays ≥ 12). Some wild-type data in Appendix Figure S1G are the same as those Appendix Figure S3B, given that the experiments were conducted simultaneously.

Data information: In **A–C**, **G**, the error bar indicates SEM. (**A**, **C**, **G**) $^{**}p < 0.01$ (Dunnett's test). (**B**) NS indicates Not Significant [unpaired *t*-test (Welch)].

# Appendix Figure S2

A

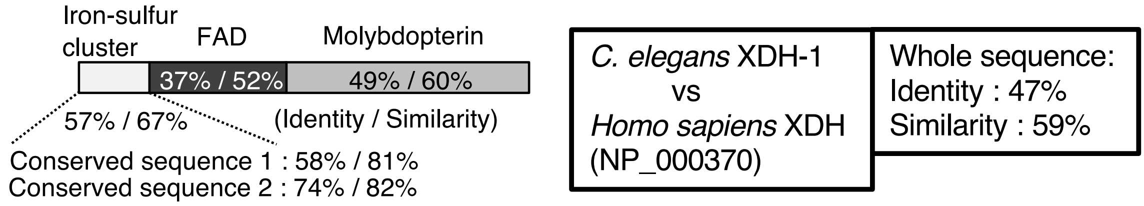


B

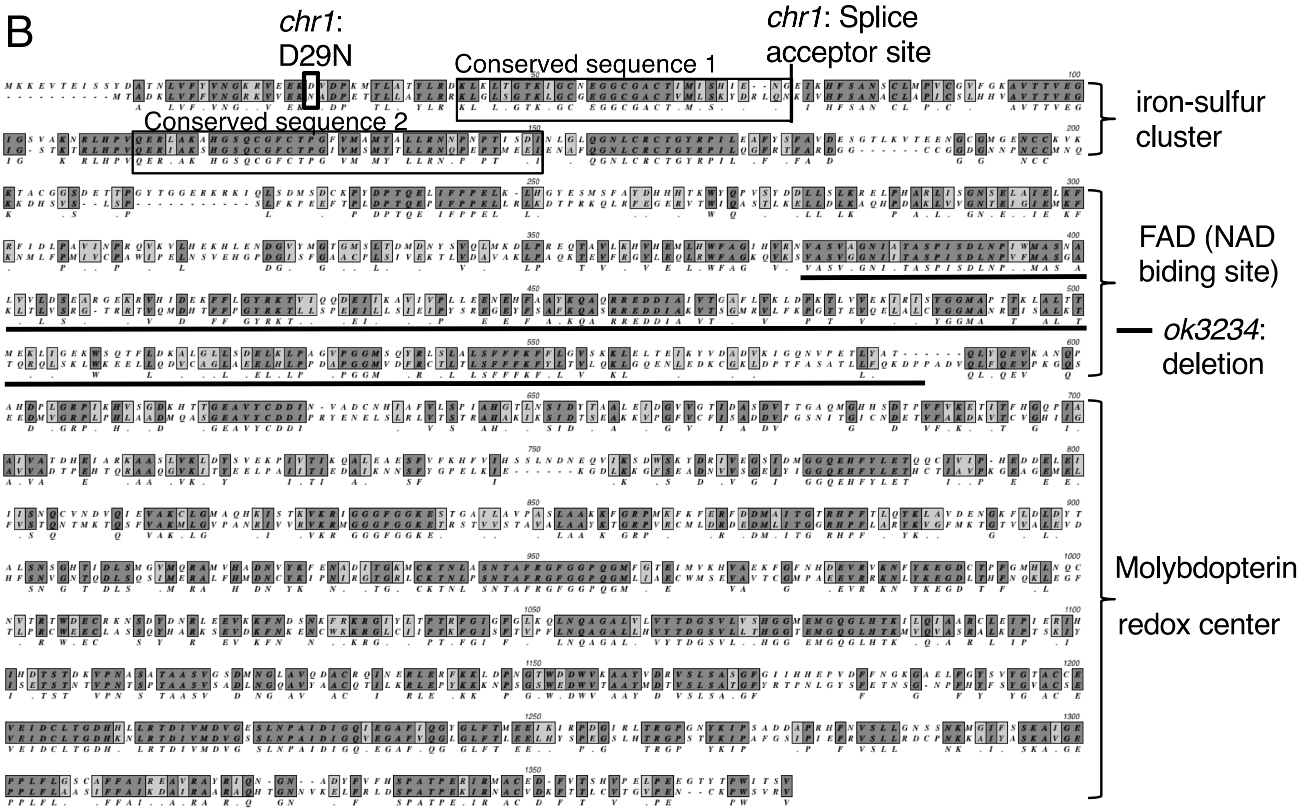


C

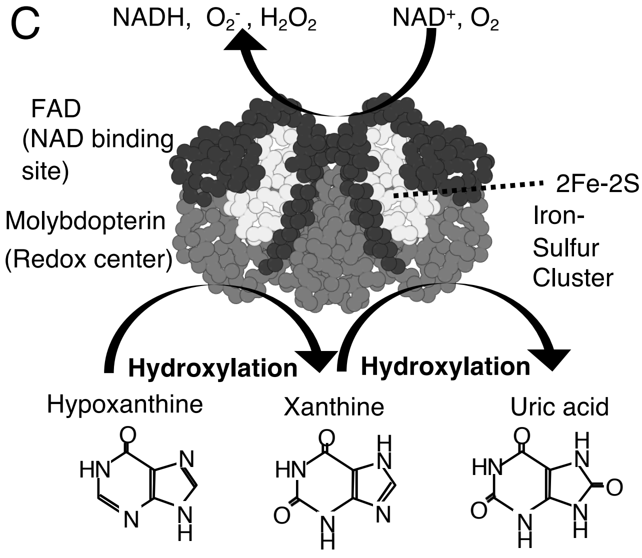

**Appendix Figure S2. XDH-1 homologous to xanthine dehydrogenase.**

(**A**) XDH-1 is homologous to human xanthine dehydrogenase (XDH). Each color represents a different xanthine dehydrogenase domain. The amino acid identity and similarity between XDH-1 and human XDH (NP_000370) are provided for each domain.

(**B**) Comparisons between XDH-1 and human XDH (NP_000370). Identical residues (dark gray highlight) and similar residues (gray highlight). The iron-sulfur cluster domains have highly conserved sequences (conserved sequences 1 and 2). *chr1* mutants possess two mutations: the first chr1 mutation results in substitution of a D to N at amino acid residue 29 and the second one is a splice site mutation. *ok3234* deletion mutation is also indicated.

(**C**) A schematic diagram of xanthine dehydrogenase (XDH). The XDH protein contains iron-sulfur clusters, FAD, and molybdopterin domains, and exists as a dimer. XDH converts hypoxanthine to xanthine, and also oxidizes xanthine to urate in the purine base salvage pathway. The colors of each sphere correspond to the bar patterns marked by domains in **A**.

## Appendix Figure S3

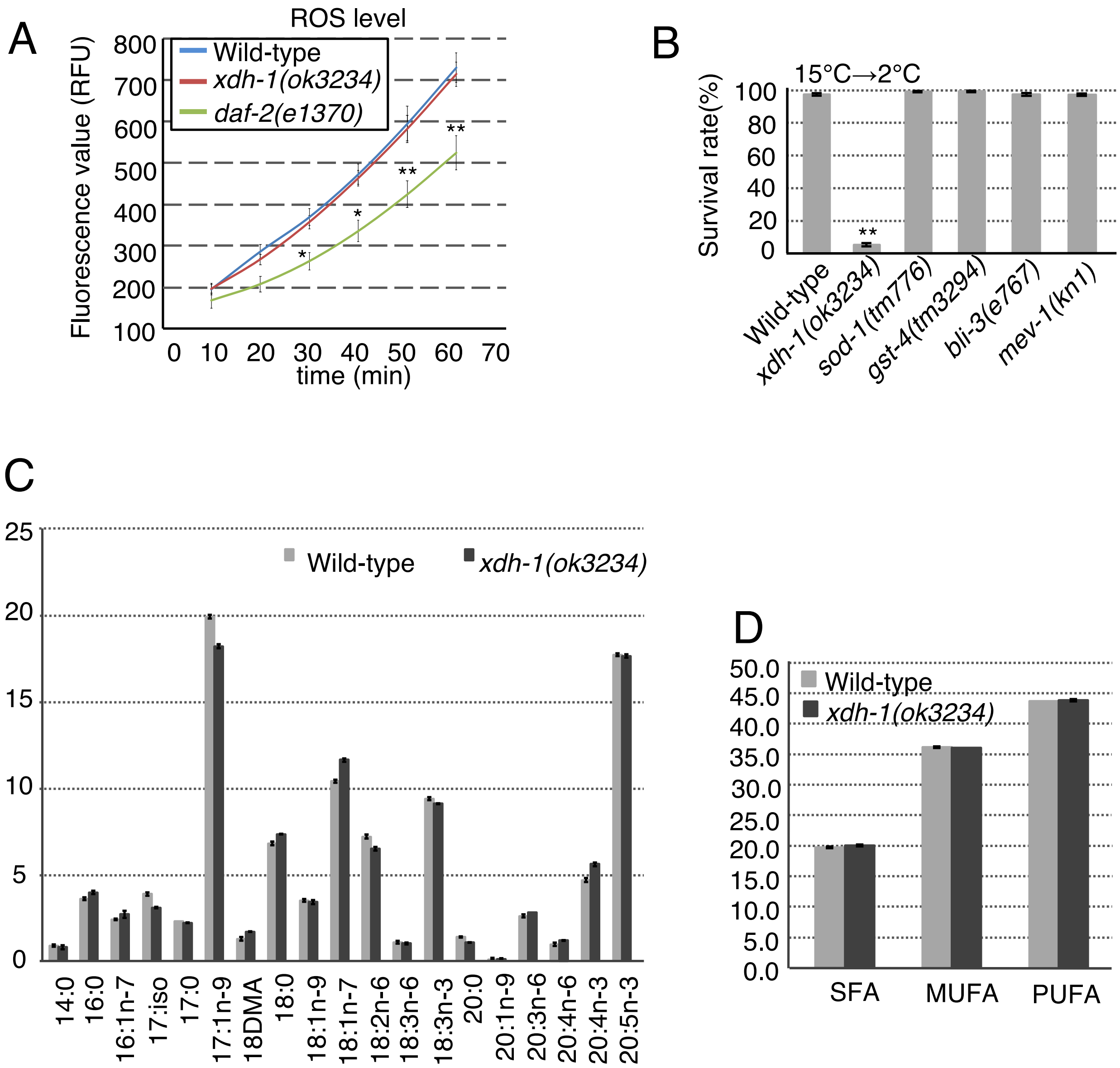


**Appendix Figure S3. ROS levels and fatty acid composition in *xdh-1* mutants.**

(**A**) Measurement of ROS levels in each animal using the fluorescent ROS indicator molecule 2′,7′-dichlorodihydrofluoroscein diacetate ($H_2$DCF-DA) (number of assays ≥ 10).

(**B**) ROS mutants exhibited normal cold tolerance (number of assays ≥ 11). Wild-type data shown in Appendix Figure S3B are also shown in Appendix Figure S1G, given that the experiments were conducted simultaneously.

(**C**, **D**) Fatty acid composition of wild-type and *xdh-1(ok3234)* total lipids. Comparison of (**C**) chain length and (**D**) saturated fatty acids (SFA), mono-unsaturated fatty acids (MUFA), and poly-unsaturated fatty acids (PUFA). N=3 for each assay.

Data information: In **A**–**D**, the error bars indicate SEM. (**A, B**) *$p < 0.05$; **$p < 0.01$ (Dunnett's test).

# Appendix Figure S4

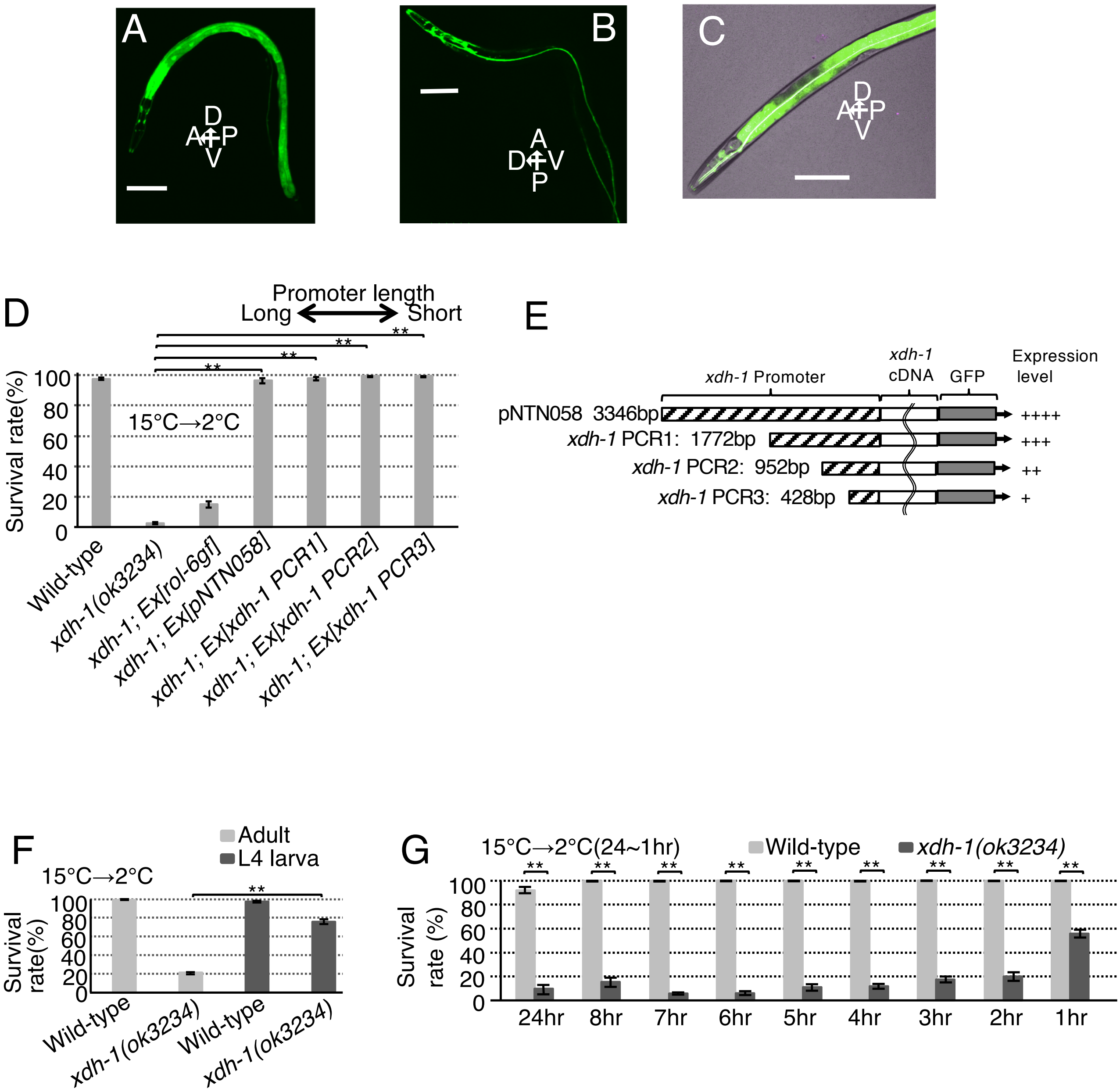


**Appendix Figure S4. Various analysis of XDH-1 and *xdh-1* mutant.**

(**A**) Wild-type with GFP expression driven by the *xdh-1* promoter (green). Scale bar: 100 μm.

(**B**) Wild-type expressing GFP driven by the *xdh-1* promoter (green). Fluorescence was undetectable in intestinal cells, most likely due to mosaicism. Excretory cell soma,

anterior excretory canal, and posterior excretory canal were observed clearly. Scale bar: 100 μm.

(**C**) Wild-type expressing GFP driven by the *xdh-1* promoter (green), excretory cell-specific expression of dsRedm (magenta), and co-expression of GFP and dsRedm in excretory cells (white). Scale bar: 100 μm.

(**D**) Cold tolerance of transgenic *xdh-1* mutant strains expressing *xdh-1 cDNA* fused with GFP driven by four lengths of *xdh-1* promoter (number of assays ≥ 18). Promoter length details are described in **E**.

(**E**) The expression of *xdh-1 cDNA* fused with GFP was driven by four lengths of *xdh-1* promoter, 428, 952, 1,772, and 3,346 bp, as indicated by diagonal lines. The fluorescence of GFP gradually decreased as the promoter length decreased, as indicated by "+." The expression patterns were almost identical for all four promoter lengths.

(**F**) Cold tolerance experiments using worms at adult and L4 larval stages (number of assays ≥ 6).

(**G**) Half of *xdh-1* mutant animals were killed by 1-h cold stimulus (number of assays ≥ 11).

Data information: In **D, F-G**, the error bars indicate SEM. (**D, F**) $**p < 0.01$ (Tukey–Kramer). (**G**) $**p < 0.01$ [unpaired *t*-test (Welch)].

# Appendix Figure S5

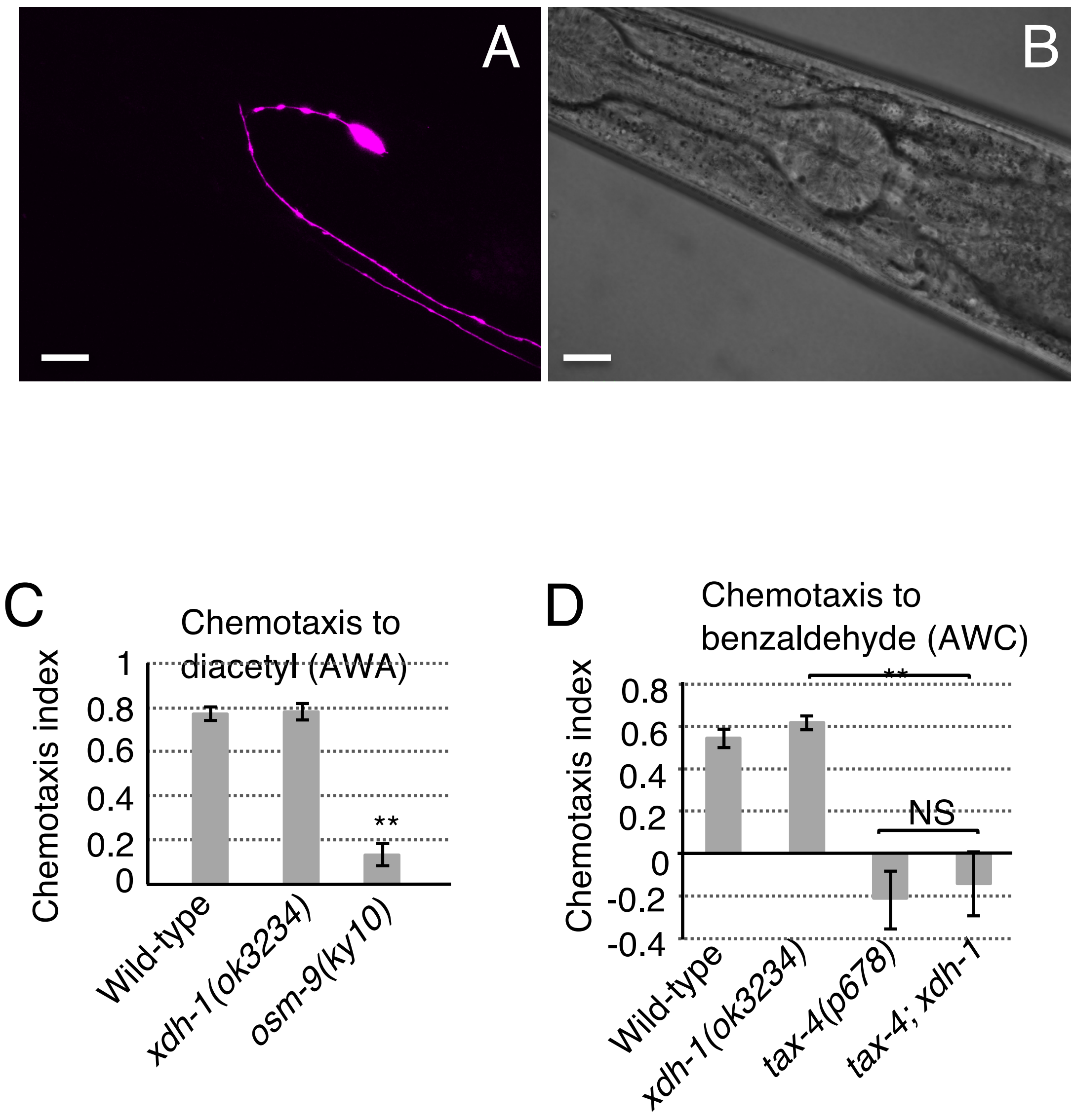


**Appendix Figure S5. AWA and AWC-mediated chemotactic responses in *xdh-1* mutants.**

(**A**, **B**) Fluorescence was observed in AVJ specifically in the wild-type animals expressing dsRedm driven by *hlh-34* promoter. dsRedm (magenta) (**A**) and Nomarski (gray) (**B**) views. Scale bars: 10 µm.

(**C**) Chemotaxis towards diacetyl sensed by AWA neurons. *xdh-1* mutants showed normal chemotaxis to diacetyl (number of assays ≥ 9).

(**D**) Chemotaxis towards benzaldehyde sensed by AWC neurons. *xdh-1* mutants showed normal chemotaxis to benzaldehyde (number of assays ≥ 12).

Data information: In **C** and **D**, the error bars indicate SEM. (**C**) $^{**}p < 0.01$ (Dunnett's test). (**D**) $^{**}p < 0.01$ (Tukey–Kramer).

# Appendix Figure S6

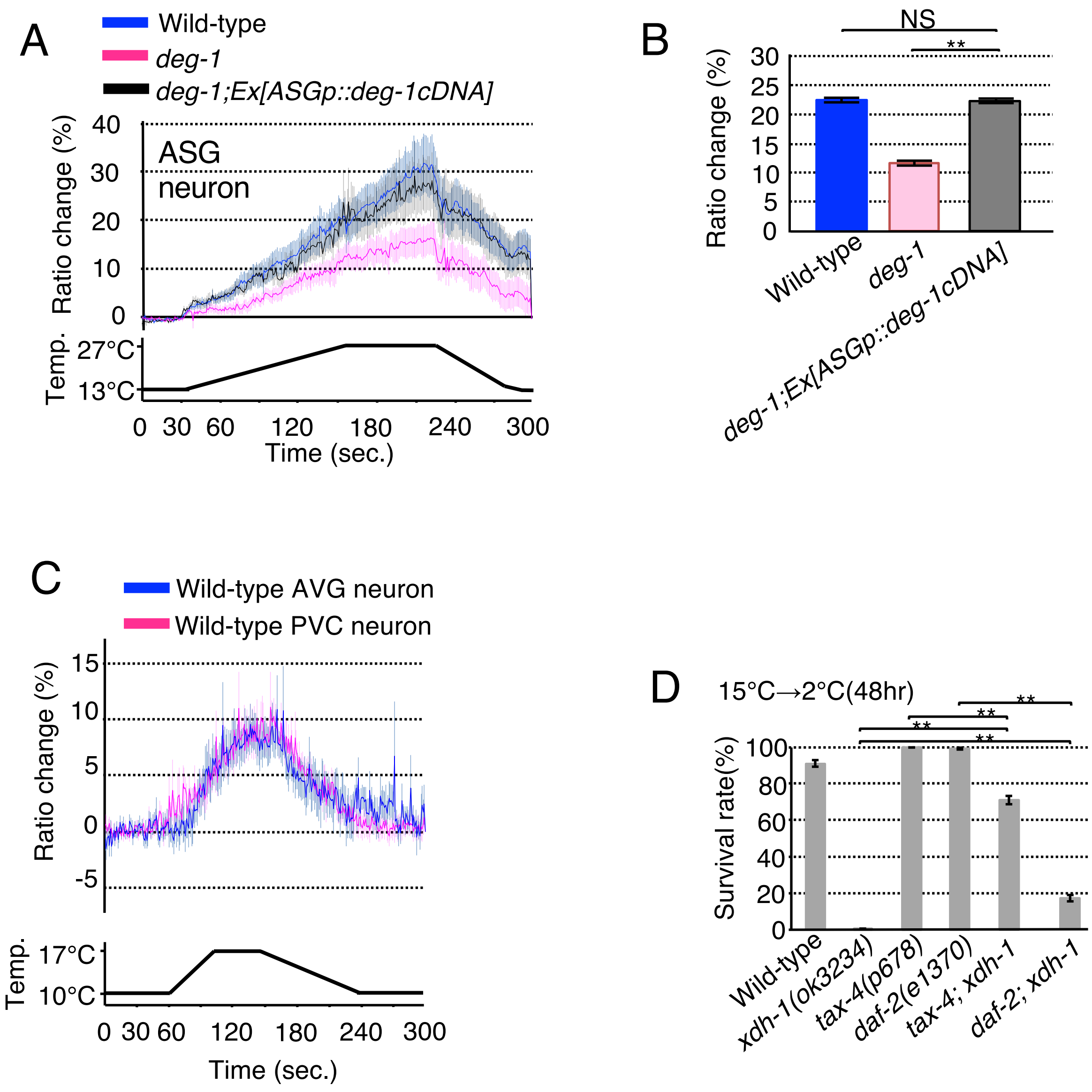


**Appendix Figure S6. Neurons expressing DEG-1 responded to temperature changes.**

(**A**, **B**) $Ca^{2+}$ imaging of ASG in animals. Bar graph, average change in cyan-yellow fluorescence ratio within 11 s from 230 to 241 s ($n \geq 20$ worms for each group). Bar graph color key is the same as that of the corresponding response curve in panel A (**B**).

(**C**) $Ca^{2+}$ imaging of AVG and PVC in the wild-type. Each graph represents the average response to temperature stimulus ($n \geq 7$ worms for each group).

(**D**) Cold tolerance assays for mutated TAX-4 cGMP-gated channel and DAF-2 insulin receptor (number of assays ≥ 16).

Data information: In **A–D**, the error bars indicate SEM. (**B, D**) $^{**}p < 0.01$ (Tukey–Kramer). In **A** and **C**, $Ca^{2+}$ imaging was performed using yellow cameleon 3.60.

# Appendix Figure S7

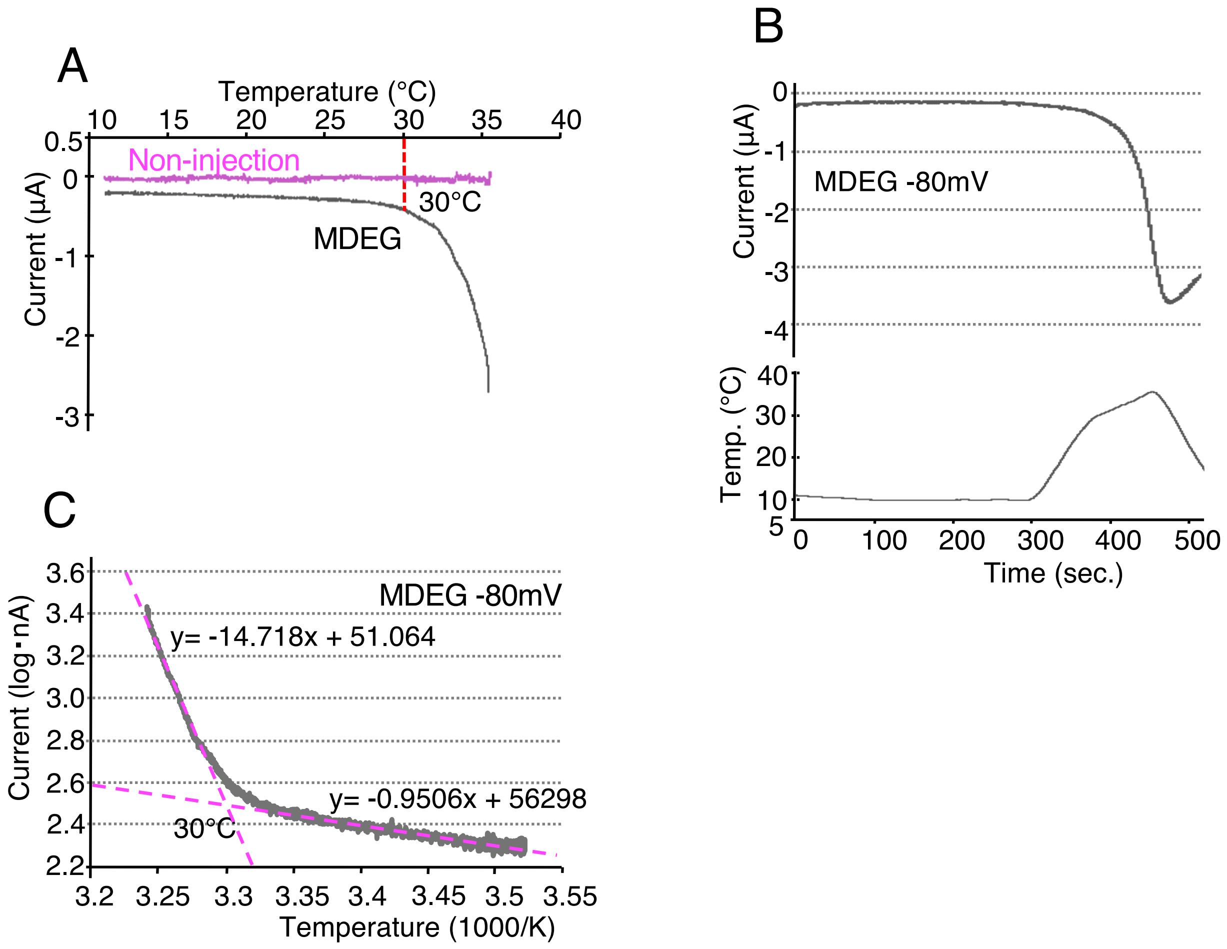


**Appendix Figure S7. Warm-sensitivity of human mechanoreceptor MDEG.**

(**A**–**C**) Responses (representative current traces) to thermal stimulus in *Xenopus* oocytes expressing MDEG.

(**A**) Heating phase relationship between current and temperature shown in **B**. Data from noninjected oocytes (n = 8 oocytes for each group).

(**B**) Representative current (upper) and temperature (lower) traces (n = 8 oocytes).

(**C**) Arrhenius plots from the data in **A**. Data obtained from the temperature increase are shown in **B**. The temperature threshold was determined by the intersection of the two extended lines shown in magenta (n = 8 oocytes).

# Appendix Figure S8

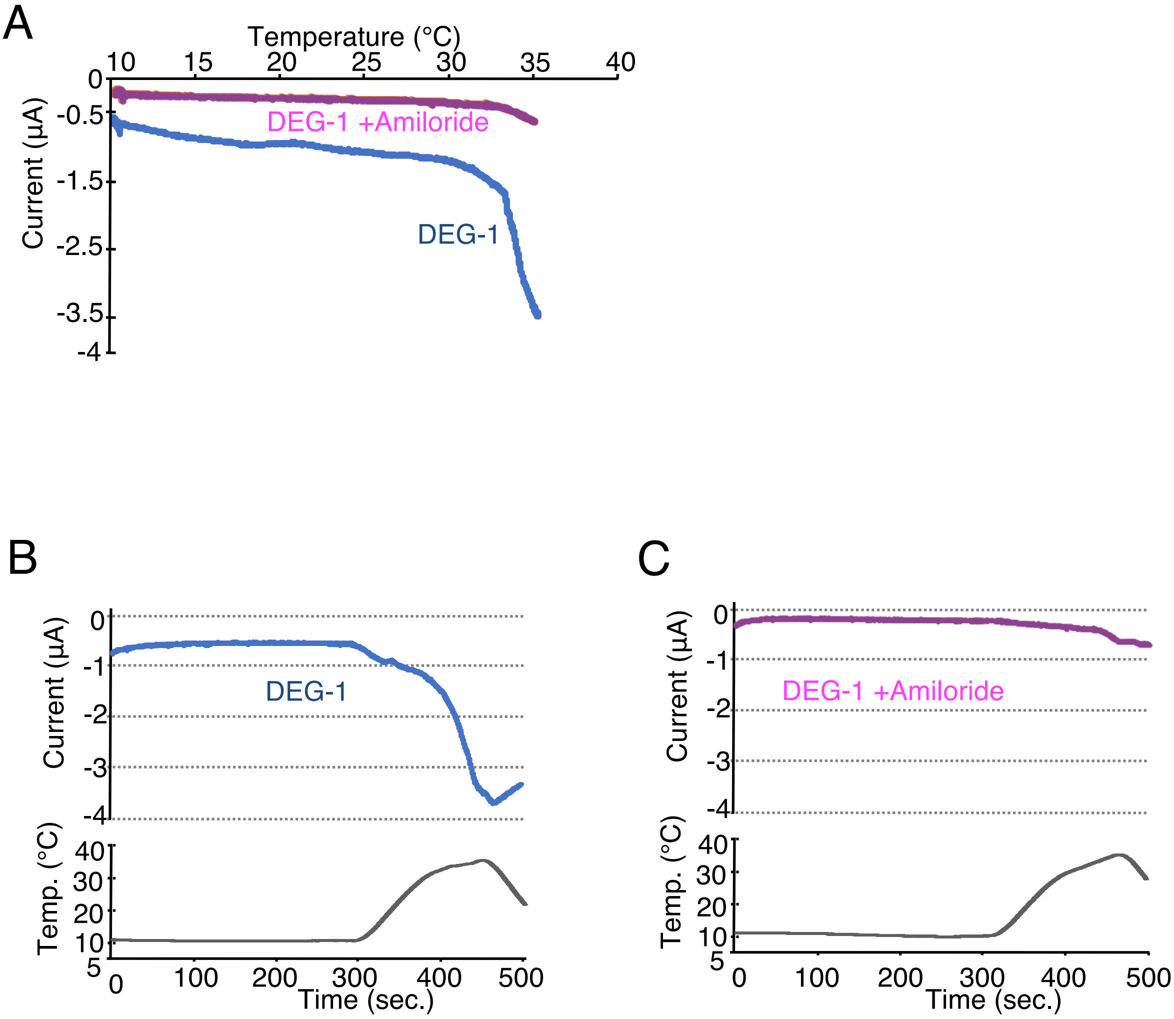


**Appendix Figure S8. Amiloride-sensitive currents in DEG-1-expressing oocytes.**

(**A**–**C**) Reactions (representative current traces) to thermal stimulus in *Xenopus* oocytes expressing DEG-1 by incubation in bath solution with an inhibitor of $Na^+$ ion channel, amiloride.

(**A**) Heating phase relationship between current and temperature shown in **B** and **C** (n = 5 oocytes for each group).

(**B**, **C**) Representative current (upper) and temperature (lower) traces (n = 5 oocytes).

# Appendix Table S1

Table S1. Tissue specific rescue experiment in Fig. 1J

| Strain | 15°C→2°C Survival rate (%) | *xdh-1cDNA* expressed in |
|---|---|---|
| Wild-type | 94.0±1.2 | / |
| *xdh-1(ok3234)* | 1.7±0.3 | / |
| *xdh-1;Ex[unc-14p::xdh-1cDNA]* | 64.7±3.4 | Almost all neurons |
| *xdh-1;Ex[pgp-12p::xdh-1cDNA]* | 9.7±2.3 | Excretory cell |
| *xdh-1;Ex[ges-1p::xdh-1cDNA]* | 4.3±1.1 | Intestine |
| *xdh-1;Ex[xdh-1p::xdh-1cDNA]* | 82.4±2.9 | Some head neurons, Excretory cell, Intestine |

# Appendix Table S2

Table S2. Tissue specific rescue experiment in Fig. 2A

| Strain | 15°C→2°C Survival rate (%) | Promoter | *xdh-1cDNA* expressed in various neurons |
|---|---|---|---|
| Wild-type | 98.6±0.3 | / | / |
| *xdh-1(ok3234)* | 3.8±0.4 | / | / |
| *xdh-1;Ex25* | 81.0±3.0 | *unc-14p* | Almost all neurons |
| *xdh-1;Ex26* | 8.1±1.5 | *dat-1p* | ADE, CEP, PDE |
| *xdh-1;Ex28* | 7.7±1.8 | *osm-6p* | ADE, ADF, ADL, AFD, AQR, ASE, ASG ASH, ASI, ASJ, ASK, AWA, AWB, AWC CEP, FLP, PDE, PHA, PHB, PQR, CEM |
| *xdh-1;Ex29* | 28.5±5.4 | *osm-6p, ncs-1p glr-1p, unc-8p* | ADE, ADF, ADL, AFD, AIB, AIN, AIY, AIZ AQR, ASE, ASG, ASH, ASI, ASJ, ASK, AVA AVB, AVD, AVE, AVG, AVJ, AVK, AWA AWB, AWC, BAG, BDU, CEP, DA, DB, DD DVC, FLP, HSN, PDA, PDB, PDE, PHA PHB, PVC, PVM, PVQ, RIG, RIM, RIS, RMD RME, RMG, SAB, SMD, URY, VA, VB, VC VD, PQR |
| *xdh-1;Ex34* | 5.9±2.1 | *unc-47p, acr-2p* | AVL, DA, DB, DD, DVB, IL1, PVQ, RIS RMD, RME, VA, VB, VD |
| *xdh-1;Ex35* | 54.5±3.4 | *osm-6p, ncs-1p glr-1p, unc-8p unc-47p, acr-2p eat-4p, unc-42p* | ADA, ADE, ADF, ADL, AFD, AIB, AIN, AIY AIZ, ALM, AQR, ASE, ASG, ASH, ASI, ASJ ASK, AUA, AVA, AVB, AVD, AVE, AVG AVJ, AVK, AVL, AVM, AWA, AWB, AWC BAG, BDU, CEP, DA, DB, DD, DVB, DVC FLP, HSN, IL1, LUA, NSM, OLL, OLQ, PDA PDB, PDE, PHA, PHB, PLM, PVC, PVD PVM, PVQ, PVR, RIG, RIM, RIS, RIV, RMD RME, RMG, SAA, SAB, SIB, SMD, URY, VA VB, VC, VD, PQR, CEM |
| *xdh-1;Ex36* | 72.2±6.5 | *eat-4p, unc-42p* | ADA, AFD, AIN, ALM, ASH, ASK, AUA, AVA AVD, AVE, AVH, AVJ, AVK, AVM, AWC, DD FLP, IL1, LUA, NSM, OLL, OLQ, PLM, PVD PVR, RIV, RMD, SAA, SIB, SMD |
| *xdh-1;Ex37* | 14.1±1.9 | *unc-86p* | ADA, AIM, AIZ, ALM, ALN, BDU FLP, HSN, IL2, NSM, PLM, URY |
| *xdh-1;Ex38* | 41.3±6.5 | *eat-4p* | ADA, AFD, AIN, ALM, ASH, ASK, AUA, AVJ AVM, AWC, FLP, IL1, LUA, NSM, OLL, OLQ PLM, PVD, PVR, |
| *xdh-1;Ex39* | 59.3±6.2 | *unc-42p* | AIN, ASH, AVA, AVD, AVE, AVH, AVJ, AVK DD, RIV, RMD, SAA, SIB, SMD |

# Appendix Table S3

Table S3. Tissue specific rescue experiment in Fig. 2B

| Strain | 15°C→2°C Survival rate (%) | Promoter | *xdh-1cDNA* expressed in various neurons |
|---|---|---|---|
| Wild-type | 92.3±1.1 | / | / |
| *xdh-1(ok3234)* | 6.4±1.4 | / | / |
| *xdh-1;Ex40* | 5.5±1.1 | *ocr-4p* | OLQ |
| *xdh-1;Ex42* | 58.5±5.1 | *ceh-10p, sra-6p* | AIN, AIY, ALA, AVJ, CEP, ASH, ASI, PVQ RID, RME |
| *xdh-1;Ex43* | 4.2±1.2 | *ocr-4p, lim-4p* | AWB, OLQ, RID, RIV, RMD, RME, SAA, SIA |
| *xdh-1;Ex45* | 3.2±2.0 | *ser-2p, ocr-4p lim-4p* | AWB, OLL, OLQ, PVD, RID, RIV, RMD RME, SAA, SIA |
| *xdh-1;Ex46* | 66.5±7.4 | *ceh-10p* | AIN, AIY, ALA, AVJ, CEP, RID, RME |
| *xdh-1;Ex47* | 11.3±1.5 | *sra-6p* | ASH, ASI, PVQ |
| *xdh-1;Ex51* | 15.5±2.3 | *glr-1p* | AIB, AVA, AVB, AVD, AVE, AVG, AVJ, DVC PVC, PVQ, RIG, RIM, RIS, RMD, RME SMD, URY |
| *xdh-1;Ex52* | 76.5±2.3 | *inx-17p, hlh-34p* | AIN, AVJ, DVA(early larva), DVC(early larva) PVT(early larva) |
| *xdh-1;Ex53* | 14.0±2.3 | *inx-17p* | AIN, DVA(early larva), DVC(early larva) PVT(early larva) |
| *xdh-1;Ex54* | 13.4±1.7 | *hlh-34p* | AVJ |
| *xdh-1;Ex63* | 8.0±1.8 | *hlh-34p, ges-1p* | AVJ, intestine |
