# Supplementary material for: The mechanoreceptor DEG‐1 regulates cold tolerance in Caenorhabditis elegans
Source: EMBO Rep. 2020 Feb 3;21(3):e48671. doi: 10.15252/embr.201948671 (PMC7054665; doi:10.15252/embr.201948671)
Supplement: Supplementary file 3 — Review Process File [file EMBR-21-e48671-s007.pdf]

EMBO *reports*

A conserved ATG2-GABARAP family interaction is critical for phagophore formation

Mihaela Bozic, Luuk van den Bekerom, Beth A. Milne, Nicola Goodman, Lisa Roberston, Alan R. Prescott, Thomas J. Macartney, Nina Dawe& David G. McEwan

Review timeline:

Submission date:	3 May 2019
Editorial Decision:	3 June 2019
Revision received:	18 October 2019
Editorial Decision:	5 December 2019
Revision received:	16 December 2019
Accepted:	23 December 2019

Editor: Martina Rembold

Transaction Report:

(Note: With the exception of the correction of typographical or spelling errors that could be a source of ambiguity, letters and reports are not edited. The original formatting of letters and referee reports may not be reflected in this compilation.)

1st Editorial Decision 3 June 2019

Thank you for the submission of your research manuscript to our journal. We have now received the full set of referee reports that is copied below.

As you will see, the referees acknowledge that the findings are potentially interesting but they also have a number of suggestions for how the study should be strengthened, and I think that all of them should be addressed. In particular, the requirement of the Atg2-GABARAP interaction for phagophore closure should be substantiated with additional experimental evidence such as EM. Moreover, the relationship or potential competition between GABARAP and WIPI4 binding should be analysed more rigorously.

Given these constructive comments, we would like to invite you to revise your manuscript with the understanding that the referee concerns (as detailed above and in their reports) must be fully addressed and their suggestions taken on board. Please address all referee concerns in a complete point-by-point response. Acceptance of the manuscript will depend on a positive outcome of a second round of review. It is EMBO reports policy to allow a single round of revision only and acceptance or rejection of the manuscript will therefore depend on the completeness of your responses included in the next, final version of the manuscript.

Revised manuscripts should be submitted within three months of a request for revision; they will otherwise be treated as new submissions. Please contact us if a 3-months time frame is not sufficient for the revisions so that we can discuss the revisions further.

When submitting your revised manuscript, please carefully review the instructions that follow below. Failure to include requested items will delay the evaluation of your revision.

1) a .docx formatted version of the manuscript text (including legends for main figures, EV figures and tables). Please make sure that the changes are highlighted to be clearly visible.

2) individual production quality figure files as .eps, .tif, .jpg (one file per figure).

3) a .docx formatted letter INCLUDING the reviewers' reports and your detailed point-by-point responses to their comments. As part of the EMBO Press transparent editorial process, the point-by-point response is part of the Review Process File (RPF), which will be published alongside your paper.

4) a complete author checklist, which you can download from our author guidelines (<http://embor.embopress.org/authorguide>). Please insert information in the checklist that is also reflected in the manuscript. The completed author checklist will also be part of the RPF.

5) Please note that all corresponding authors are required to supply an ORCID ID for their name upon submission of a revised manuscript (<https://orcid.org/>). Please find instructions on how to link your ORCID ID to your account in our manuscript tracking system in our Author guidelines (<http://embor.embopress.org/authorguide>).

6) We replaced Supplementary Information with Expanded View (EV) Figures and Tables that are collapsible/expandable online. A maximum of 5 EV Figures can be typeset. EV Figures should be cited as 'Figure EV1, Figure EV2" etc... in the text and their respective legends should be included in the main text after the legends of regular figures.

- For the figures that you do NOT wish to display as Expanded View figures, they should be bundled together with their legends in a single PDF file called *Appendix*, which should start with a short Table of Content. Appendix figures should be referred to in the main text as: "Appendix Figure S1, Appendix Figure S2" etc. See detailed instructions regarding expanded view here: <http://embor.embopress.org/authorguide#expandedview>.

- Additional Tables/Datasets should be labeled and referred to as Table EV1, Dataset EV1, etc. Legends have to be provided in a separate tab in case of .xls files. Alternatively, the legend can be supplied as a separate text file (README) and zipped together with the Table/Dataset file.

7) We would also encourage you to include the source data for figure panels that show essential data. Numerical data should be provided as individual .xls or .csv files (including a tab describing the data). For blots or microscopy, uncropped images should be submitted (using a zip archive if multiple images need to be supplied for one panel). Additional information on source data and instruction on how to label the files are available
<http://embor.embopress.org/authorguide#sourcedata>.

8) Regarding data quantification, please ensure to specify the name of the statistical test used to generate error bars and P values, the number (n) of independent experiments underlying each data point (not replicate measures of one sample), and the test used to calculate p-values in each figure legend. Discussion of statistical methodology can be reported in the materials and methods section, but figure legends should contain a basic description of n, P and the test applied.
Please note that error bars and statistical comparisons may only be applied to data obtained from at least three independent biological replicates.
Please also include scale bars in all microscopy images.

We would also welcome the submission of cover suggestions, or motifs to be used by our Graphics Illustrator in designing a cover.

As part of the EMBO publication's Transparent Editorial Process, EMBO reports publishes online a Review Process File to accompany accepted manuscripts. This File will be published in conjunction with your paper and will include the referee reports, your point-by-point response and all pertinent correspondence relating to the manuscript.

You are able to opt out of this by letting the editorial office know (emboreports@embo.org). If you do opt out, the Review Process File link will point to the following statement: "No Review Process File is available with this article, as the authors have chosen not to make the review process public

in this case."

I look forward to seeing a revised version of your manuscript when it is ready. Please let me know if you have questions or comments regarding the revision.

****

REFEREE REPORTS

Referee #1:

This manuscript from the McEwan lab addresses the role of ATG2A/B. ATG2 is one of the less well-studied ATG proteins and there is significant interest in this protein, and understanding its function. This manuscript reveals an interaction of ATG2A and B with GABARAPs via a LC3-interacting motif (LIR). The LIR motif is close to the interaction motif previously known to modulate interaction between ATG2 and WIPI4. The LIR identified in GABARAP is highly conserved. The authors detail the requirements for recruitment of GABARAP and WIPI4 in a set of well-controlled experiments concluding the ATG2-GABARAP interaction is required for flux, but ATG2-WIPI4 is dispensable. Next, they test the requirements for these interactions for autophagosome closure. The confirmed previous work using the ATG2DKO that there was aberrant formation, and also ATG9 distribution is altered. These phenotypes were rescued by wild-type and YFS ATG2, as was recruitment of Syntaxin 17. The LIR mutant however was unable to rescue any phenotype, and produced protease-sensitive autophagosomes which did not recruit Syntaxin 17. Overall, the data is well presented, convincing and of interest to the field. There are a few points to address:
Major points:
1. It would be informative to understand how much GABARAP-ATG2 complex is required to recruit detectable amounts of WIPI4 and to more rigorously test the competition between the LIR and YFS. In Figure 2, IP GFP-GABARAP and probe for WIPI4 with and without ATG2 WT and mLIR by titrating the amount of ATG2.
2. Can the authors IP WIPI4 and detect ATG2A, ATG2B or GABARAP?
3. Figure 2E quantify the pulldowns as the input is uneven, in particular the WIPI4, and the amount of HA-ATG2 mLIR in the IP is less than YFS. Do the authors have evidence that WIPI4 levels are increased in starvation?
4. It would be informative to check the localization of WIPI4 in the ATG2 WT, mLIR and YFS mutants under the conditions used to provide more information about the interaction of the ATG2, GABARAP and WIPI4 complexes.
Minor points:
Clarify the following statements:
-page 6 line 14 "functional consequences and in vivo preferences directed towards GABARAP" - what about interaction with LC3A? what functional consequences are shown in Figure 2A that the authors are referring to?
-page 7 line 1-2 this sentence is confusing "It remains to be seen....autophagy." Isn't this what the authors are trying to address?

Referee #2:

In this paper, McEwan and colleagues identified a motif in mammalian ATG2 for interaction with GABARAP. This motif is in close proximity to the ATG2-WIPI4 interaction site. They present data indicating that ATG2-GABARAP interaction mutants are unable to close phagophores, resulting in blocked autophagy, similar to ATG2A/B double KO cells. In contrast, the ATG2-WIPI4 interaction mutant restored autophagy flux and appeared to restore what the authors refer to as phagophore closure, similar to wild type ATG2.

This study has been well conducted and contains original and interesting observations. However, there are some suggestions for revisions before publication.

Major

1. The authors claim that ATG2-mLIR is critical for phagophore closure. However, far more experimental proof and methods are needed to be really convinced of authors statement and interpretations and their model for closure, as this hypothesis floats up to be the main purpose of this study.

2. In Fig2A, it would be nice to have better blots for myc-ATG2A. It's not easy to see the bands and the loss of interaction in LIR mutant compared to WT. One cannot see any band for GABARAPL1 even if it is visible in EV2A.

3. In Fig4B, it is necessary to show LC3/GABARAP blots for the prot K protection assay

Minor:

4. Some referencing needs improvement. Examples are Stx17 recruitment, etc.

5. Is it really necessary to introduce multiple terms for LIR? I'd suggest to adhere to Johansen's terminology (see reviews) and refer to these sequences as LIR.

Referee #3:

Bozic et. al identified a new role of ATG2 in phagophore closure mediated by its direct interaction with ATG8s proteins through newly identified LIR domain. This LIR domain, located 30 amino acids upstream of previously characterized WIPI4 interaction motif (YFS), represents a distinct interaction site. The authors claim that Atg2-Atg8 but not Atg2-WIPI4 interaction is needed for phagophore closure and the overall autophagic flux.
This research provides an interesting insight into newly identified ATG2 interaction with ATG8s during autophagosome biogenesis. For this study to be fully convincing, the authors' main claim concerning the importance of this interaction for autophagosomal membrane closure requires additional experimental data. Also, the relationship between the two adjacent sites for Atg2 interaction it WIPI4 and Atg8s should be better characterized. It is important for example to determine whether these interactions are mutually exclusive suggesting a competition between these factors during the process of autophagosome biogenesis.

Additional comments
Authors conclude that both ATG2A and ATG2B directly interact with GABARAP family of ATG8 proteins, but most of the data are related to ATG2A only (including reconstitution experiments in ATG2A/ATG2B double knockout).
The authors emphasis the direct interaction of Atg2 with GABARAP, but most of the experiments are concentrated on LC3B. The reconstitution experiments as well as distinguishing between phagophores and autophagosomes should be based on GABARAP too and not only LC3B.
Moreover, in order to conclude that ATG2 interact with GABARAP family, all family members should be tested.
Figure 1D and 1E - does GABARAP colocalizes to ATG2A? In panel D the data provided according to GABA-L1 only. In panel E the data present GABARAP only. Does GABA-L1 co-immunoprecipitates with ATG2A? What about GABARAP-L2?
Figure 4B - It is unclear why the authors only test protease protection in the presence of BafA but not with other way to block autophagosome-lysosome fusion (for example knockdown of syntaxin 17). Moreover, as indicated above additional tools such as EM and supper-resolution microscopy may help characterize the intermediate membranes obtained when the interaction between Atg2 and Atg8s is disrupted.
Figure EV4 - western blots are missing to support the IF data.

1st Revision - authors' response 18 October 2019

Authors response to the Referees comments.

Referee #1:

This manuscript from the McEwan lab addresses the role of ATG2A/B. ATG2 is one of the less well-studied ATG proteins and there is significant interest in this protein, and understanding its function. This manuscript reveals an interaction of ATG2A and B with GABARAPs via a LC3-interacting motif (LIR). The LIR motif is close to the interaction motif previously known to modulate interaction between ATG2 and WIPI4. The LIR identified in GABARAP is highly conserved. The authors detail the requirements for recruitment of GABARAP and WIPI4 in a set of well-controlled experiments concluding the ATG2-GABARAP interaction is required for flux, but ATG2-WIPI4 is dispensable. Next, they test the requirements for these interactions for autophagosome closure. The confirmed previous work using the ATG2DKO that there was aberrant formation, and also ATG9 distribution is altered. These phenotypes were rescued by wild-type and YFS ATG2, as was recruitment of Syntaxin 17. The LIR mutant however was unable to rescue any phenotype, and produced protease-sensitive autophagosomes which did not recruit Syntaxin 17. Overall, the data is well presented, convincing and of interest to the field. There are a few points to address:

We would like to thank the referee for their constructive comments and believe that the suggested experiments have strengthened the manuscript as a result of their inclusion.

1. It would be informative to understand how much GABARAP-ATG2 complex is required to recruit detectable amounts of WIPI4 and to more rigorously test the competition between the LIR and YFS. In Figure 2, IP GFP-GABARAP and probe for WIPI4 with and without ATG2 WT and mLIR by titrating the amount of ATG2.

As the referee has suggested, we have confirmed the reverse IP experiment that now forms part of Figure 2 (Figure 2F and quantified in Figure 2G). In summary, endogenous WIPI4 only co-immunoprecipitated with ATG2A-WT and not the mLIR or mYFS (as expected), indicating that WIPI4 does not interact directly with GABARAP but forms a complex of WIPI4-ATG2A-GABARAP.

We also used overexpression of increasing amounts of WIPI4 to assess whether we could out-compete the interaction of GFP-GABARAP with endogenous ATG2A/ATG2B. We show that indeed, having high concentrations of WIPI4 (such as by overexpression) we lose a large proportion of ATG2A and ATG2B binding to GABARAP (Figure 2H and quantified in Figure 2I). However, the reverse was not true (i.e increasing GABARAP concentration to out compete WIPI4 interaction), indicating that WIPI4 is the more dominant and stable interaction (Figure EV2J-K). This is also indicated by our ability to immunoprecipiate WIPI4 with endogenous GFP-tagged ATG2A under non-stimulated conditions and that this interaction has been previously shown to be a stable interaction in multiple studies. We believe that upon activation of autophagy, through an as yet unidentified mechanism, stimulates GABARAP recruitment and complex formation between GABARAP-ATG2A-WIPI4 for the efficient formation of autophagosomes.

2. Can the authors IP WIPI4 and detect ATG2A, ATG2B or GABARAP?

Due to the limited amounts of anti-WIPI4 antibody (a kind gift from Prof. Sharon Tooze) and its limited use for both immunoprecipitation and immunofluorescence we have been unable to perform these experiments. However, using overexpressed GFP-tagged WIPI4 we could show efficient immunoprecipitation of endogenous ATG2A and ATG2B from cells (Figure EV2J-K). However, we could not detect endogenous GABARAP/GABARAP-L1. In-light of our results where overexpression of WIPI4 can out compete GABARAP binding, this is not surprising.
We could detect an ATG2-WIPI4 coprecipitation with a limited number of mammalian ATG8s (Figure EV1D). This also follows on from work by other groups showing both an in vitro and in vivo complex between ATG2s and WIPI4 [1-4].

3. Figure 2E quantify the pulldowns as the input is uneven, in particular the WIPI4, and the amount of HA-ATG2 mLIR in the IP is less than YFS. Do the authors have evidence that WIPI4 levels are increased in starvation?

We have now quantified the co-IPs as suggested and this is now shown as Figure 2D.
As for the WIPI4 levels changing, we believe it is potential stabilisation effect that is also reflected in some of the pull downs. Notably, we see consistent differences between ATG2A/B DKO cells and those reconstituted with ATG2A WT.

4. It would be informative to check the localization of WIPI4 in the ATG2 WT, mLIR and YFS

mutants under the conditions used to provide more information about the interaction of the ATG2, GABARAP and WIPI4 complexes.

We agree with the referee on this point, however as mentioned above the antibody used to detect WIPI4 by western blot was not suitable for either immunoprecipitation or immunofluorescence as this had been tested extensively in the Tooze lab. Given our results with the overexpression of WIPI4 on GARARAP-ATG2 interaction (Figure 2H-I), using the GFP-construct to assess this was difficult and provided inconclusive results. However, the referee deems that this is required we are happy to include this in the final version. Currently, we have excluded this due to space constraints for the EMBO reports short report format.

Minor points:

Clarify the following statements:

-page 6 line 14 "functional consequences and in vivo preferences directed towards GABARAP" - what about interaction with LC3A? what functional consequences are shown in Figure 2A that the authors are referring to?

We apologise for the confusion. We have clarified the sentence to read "..however we have been unable to confirm an endogenous interaction" as we could not detect LC3A in ATG2 IPs. From our results in Figure EV1D, it appears that GABARAP and GABARAP-L1 interact more with ATG2A/B than LC3A and thus was the major focus of this paper. However, we cannot rule out a role for ATG2-LC3A interaction during the autophagy pathway.

-page 7 line 1-2 this sentence is confusing "It remains to be seen....autophagy." Isn't this what the authors are trying to address?

We agree with the referee and have removed this sentence for clarity.

Referee #2:

In this paper, McEwan and colleagues identified a motif in mammalian ATG2 for interaction with GABARAP. This motif is in close proximity to the ATG2-WIPI4 interaction site. They present data indicating that ATG2-GABARAP interaction mutants are unable to close phagophores, resulting in blocked autophagy, similar to ATG2A/B double KO cells. In contrast, the ATG2-WIPI4 interaction mutant restored autophagy flux and appeared to restore what the authors refer to as phagophore closure, similar to wild type ATG2.

This study has been well conducted and contains original and interesting observations. However, there are some suggestions for revisions before publication.

We would like to thank the referee for their constructive comments and believe that the suggested experiments have strengthened the manuscript as a result of their inclusion.

1. The authors claim that ATG2-mLIR is critical for phagophore closure. However, far more experimental proof and methods are needed to be really convinced of authors statement and interpretations and their model for closure, as this hypothesis floats up to be the main purpose of this study.

We agree with the referee on this point and would like to thank the referee for the suggestions. We have now included images from both Airyscan super resolution confocal microscopy (Figure 5 A-C Figure EV4A-B, Movie EV1A and Movie EV1B) and transmission electron microscopy (Figure 5D) to analyse the effect of the mutants on the formation of autophagosomes. This has led to us observing that the ATG2A-mLIR results in the formation of multiple, immature phagophores that cluster with ER. These remain proteinase K sensitive and are "open" similar to ATG2A/B double knockouts and consistent with observations from other groups [5-7]. We have altered our title to reflect these new conclusions as a result of the experiments suggested by the reviewers (see above).

2. In Fig2A, it would be nice to have better blots for myc-ATG2A. It's not easy to see the bands and the loss of interaction in LIR mutant compared to WT. One cannot see any band for GABARAPL1 even if it is visible in EV2A.

We agree that this was not clear. This panel has been replaced for both ATG2A and ATG2B and is now Figure EV2H.

3. In Fig4B, it is necessary to show LC3/GABARAP blots for the prot K protection assay.

We agree that the inclusion of LC3/GABARAP blots would enhance the results and be more complete. However, during the course of the studies, we found that both LC3 and GABARAP are proteinase K resistant, even in the presence of triton x-100 (see below for example (i)). This was also the case for GFP-LC3B (see below, (ii)). This is in spite of the fact that p62/SQSTM1 and HA-ATG2A were efficiently degraded and were model substrates for this assay. We hope the reviewers will take this into account and also the second part of the figure, STX17 localization, to demonstrate that the p62/LC3 structures we observe are not complete autophagosomes, but rather "open" phagophores.

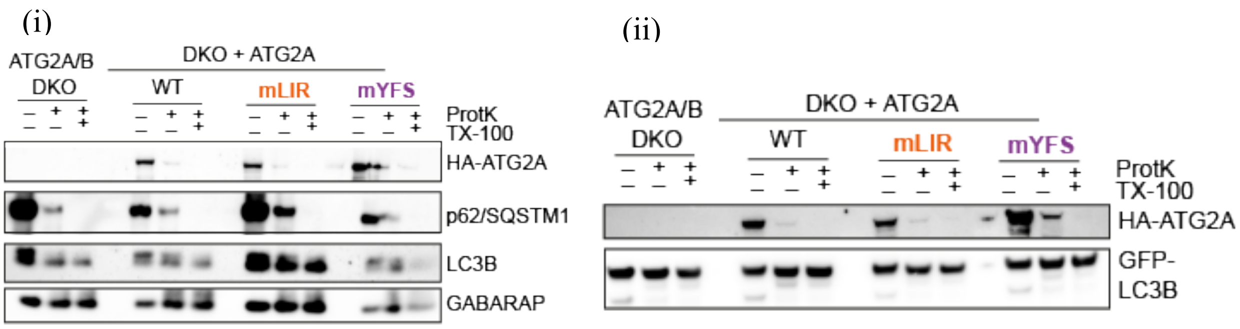

Minor:

4. Some referencing needs improvement. Examples are Stx17 recruitment, etc.

We have updated the references for STX17 recruitment accordingly (page 9 of main text).

5. Is it really necessary to introduce multiple terms for LIR? I'd suggest to adhere to Johansen's terminology (see reviews) and refer to these sequences as LIR.

We agree that multiple names for the same sequence type do not make for easier reading and have simplified the terminology and adhered to only using LIR to describe the sequences as per the referee's suggestion.

Referee #3:

Bozic et. al identified a new role of ATG2 in phagophore closure mediated by its direct interaction with ATG8s proteins through newly identified LIR domain. This LIR domain, located 30 amino acids upstream of previously characterized WIPI4 interaction motif (YFS), represents a distinct interaction site. The authors claim that Atg2-Atg8 but not Atg2-WIPI4 interaction is needed for phagophore closure and the overall autophagic flux.
This research provides an interesting insight into newly identified ATG2 interaction with ATG8s during autophagosome biogenesis. For this study to be fully convincing, the authors' main claim concerning the importance of this interaction for autophagosomal membrane closure requires additional experimental data. Also, the relationship between the two adjacent sites for Atg2 interaction it WIPI4 and Atg8s should be better characterized. It is important for example to determine whether these interactions are mutually exclusive suggesting a competition between these factors during the process of autophagosome biogenesis.

We would like to thank the referee for their constructive comments and believe that the suggested experiments have strengthened the manuscript as a result of their inclusion.

Authors conclude that both ATG2A and ATG2B directly interact with GABARAP family of ATG8 proteins, but most of the data are related to ATG2A only (including reconstitution experiments in ATG2A/ATG2B double knockout).

The referee is correct to in their observation. We can show that ATG2B interacts with GABARAP/GABARAP-L1 through a conserved LIR sequence that is shared not only with human ATG2A but multiple other species of ATG2s. It was our intention that the manuscript include both human ATG2 isoforms in order to gain a more comprehensive understanding of their biological function. However, we have been unable to reconstitute ATG2A/B double knockout cells with ATG2B WT and mutants to a satisfactory level (either too high or too low) within the timeframe of revision. This is something that we are actively working on to resolve, but unfortunately, we cannot include in this manuscript preparation. We also note that a number of the papers currently published that analyse ATG2 function, are also solely focused on ATG2A and perhaps this is a common problem with ATG2B overexpression. Where possible, we have limited our conclusions to that of the role of ATG2A but have included more in the discussion sections regarding the role of ATG2B in this process. We hope that the referee nonetheless finds our data of importance to the field.

The authors emphasis the direct interaction of Atg2 with GABARAP, but most of the experiments are concentrated on LC3B. The reconstitution experiments as well as distinguishing between phagophores and autophagosomes should be based on GABARAP too and not only LC3B.

We agree and have included GABARAP/GABARAP-L1 or pan GABARAP staining or western blot analysis throughout. These include co-precipitation experiments of GABARAP and WIPI4 with ATG2A WT and mutants (Figure 2D-I), GABARAP. Blotting on flux experiments (Figure 3C and EV4C) and importantly GABARAP and GABARAP-L1 co-staining on super resolution images of the phagophore formations in WT and mutant reconstituted cells (Figure 5C and EV4B).

Moreover, in order to conclude that ATG2 interact with GABARAP family, all family members should be tested.

We agree with the referee that all isoforms should have been tested. We have no included co-precipitation of experiments using GFP-tagged ATG8 isoforms (LC3A, LC3B, LC3C, GABARAP, GABARAP-L1 and GABARAP-L2). From this we can show that both ATG2A and ATG2B co-precipitate with GABARAP and GABARAP-L1 mainly, but also weakly with LC3A (Figure EV1D). Interestingly, we also found that WIPI4 also co-precipitated with ATG2A/B in these samples.

Figure 1D and 1E - does GABARAP colocalizes to ATG2A? In panel D the data provided according to GABA-L1 only.

We have attempted the co-localization experiments with GABARAP and CRISPR tagged GFP-ATG2A. However, the specific GABARAP antibody (Abgent, AP1821a) that has previously been used for IF studies [8] requires cells to be incubated in methanol for 5 minutes (permeabilization) in order to observe GABARAP puncta. This method is unfortunately incompatible with the endogenous ATG2A staining we show in the rest of the figure that used a much less harsh, saponin-based, permeabilization method. However, given the high degree of sequence identity between GABARAP and GABARAP-L1 (86%) and that ATG2A/B seem to interact equally well with both isoforms, we have been unable to precisely define which is the preferred interaction partner. We have updated the text throughout the manuscript to reflect this.

In panel E the data present GABARAP only.
Does GABA-L1 co-immunoprecipitates with ATG2A? What about GABARAP-L2?

As per referee 3's earlier suggestion where we tested interaction of ATG2s with all ATG8 family members, we found no or very weak interaction of ATG2A and ATG2B with GABARAP-L2 by co-precipitation from cells (Figure EV1D).

Figure 4B - It is unclear why the authors only test protease protection in the presence of BafA but not with other way to block autophagosome-lysosome fusion (for example knockdown of syntaxin 17).

We apologise for the lack of clarity in this. We used BafA1 in combination with starvation to simply accumulate autophagy vesicles (phagophores/autophagosomes/autolysosomes that would contain (or not in the case of DKO and DKO+2A-mLIR) p62/SQSTM1 that we could test using the limited proteolysis assay. Knockdown of syntaxin17 would also have been an option, however this would have had to also be combined with knockdown of YKT6 to

completely block fusion. Using a chemical inhibitor of degradation, we were able simply and reproducibly accumulate the vesicles of interest.

Moreover, as indicated above additional tools such as EM and supper-resolution microscopy may help characterize the intermediate membranes obtained when the interaction between Atg2 and Atg8s is disrupted.

As per referee 3 and also referee 2's suggestion, we have now included images from both Airyscan super resolution confocal microscopy (Figure 5 A-C Figure EV4A-B, Movie EV1A and Movie EV1B and transmission electron microscopy (Figure 5D) to analyse the effect of the mutants on the formation of autophagosomes. This has led to us observing that the ATG2A-mLIR results in the formation of multiple, immature phagophores that cluster with ER. These remain proteinase K sensitive and are "open" similar to ATG2A/B double knockouts and consistent with observations from other groups [5-7]. We have altered our title to reflect these new conclusions as a result of the experiments suggested by the reviewers (see above).

Figure EV4 - western blots are missing to support the IF data.

As per the reviewer's suggestion, we have included the western blot data to support the IF data (Figure EV4C).

References:

1. Zheng JX, Li Y, Ding YH, Liu JJ, Zhang MJ, Dong MQ, Wang HW, Yu L (2017) Architecture of the ATG2B-WDR45 complex and an aromatic Y/HF motif crucial for complex formation. *Autophagy* 13: 1870-1883
2. Chowdhury S, Otomo C, Leitner A, Ohashi K, Aebersold R, Lander GC, Otomo T (2018) Insights into autophagosome biogenesis from structural and biochemical analyses of the ATG2A-WIPI4 complex. *Proceedings of the National Academy of Sciences of the United States of America* 115: E9792-E9801
3. Stanga D, Zhao Q, Milev MP, Saint-Dic D, Jimenez-Mallebrera C, Sacher M (2019) TRAPPC11 functions in autophagy by recruiting ATG2B-WIPI4/WDR45 to preautophagosomal membranes. *Traffic*
4. Bakula D, Muller AJ, Zuleger T, Takacs Z, Franz-Wachtel M, Thost AK, Brigger D, Tschan MP, Frickey T, Robenek H, *et al.* (2017) WIPI3 and WIPI4 beta-propellers are scaffolds for LKB1-AMPK-TSC signalling circuits in the control of autophagy. *Nat Commun* 8: 15637
5. Tamura N, Nishimura T, Sakamaki Y, Koyama-Honda I, Yamamoto H, Mizushima N (2017) Differential requirement for ATG2A domains for localization to autophagic membranes and lipid droplets. *FEBS Lett* 591: 3819-3830
6. Velikkakath AK, Nishimura T, Oita E, Ishihara N, Mizushima N (2012) Mammalian Atg2 proteins are essential for autophagosome formation and important for regulation of size and distribution of lipid droplets. *Mol Biol Cell* 23: 896-909
7. Kishi-Itakura C, Koyama-Honda I, Itakura E, Mizushima N (2014) Ultrastructural analysis of autophagosome organization using mammalian autophagy-deficient cells. *J Cell Sci* 127: 4089-102
8. Joachim J, Razi M, Judith D, Wirth M, Calamita E, Encheva V, Dynlacht BD, Snijders AP, O'Reilly N, Jefferies HBJ, *et al.* (2017) Centriolar Satellites Control GABARAP Ubiquitination and GABARAP-Mediated Autophagy. *Curr Biol* 27: 2123-2136 e7

2nd Editorial Decision 5 December 2019

Thank you for your patience while we have reviewed your revised manuscript. Unfortunately, we have not received the report from former referee #2 but since referee #1 and #3 are very positive about the study and support publication in EMBO reports, I have decided to proceed.

I am therefore writing with an 'accept in principle' decision, which means that I will be happy to accept your manuscript for publication once a few minor issues/corrections have been addressed, as follows.

1) Our data editors from Wiley have already inspected the Figure legends for completeness and accuracy. Please see the required changes in the attached Word file.

2) Author contributions: please specify the contribution of Beth Milne and Lisa Robertson

3) Movies: please remove their legends from the main manuscript file and supply them as simple README.txt file. Then zip the movie with its legend and upload the .zip file.

4) Please remove the list of abbreviations from the front page. All abbreviations have to be explained in the text.

5) Please convert the table with plasmids that is currently in Materials and Methods to "Table 2", move it to the end of the manuscript and provide a callout in the text.

6) Table 1 contains red color, which unfortunately cannot be typeset. Please choose another highlight, e.g., underline or bold.

7) Source data: Thank you for providing it. Could you please double-check the data supplied for Figure 2F? You note that the higher exposure for GFP-GABARAP was not used for the publication but it appears to be the scan that was used. Moroever, what is labeled GFP-GABARAP input in the source data file appears as IP in the figure panel. Please double-check this.

8) We noticed that the resolution of Western blot images is generally rather low throughout the paper. If you happen to have higher resolution images/scans of your blots, please update the panels. If the panels already display the best resolution you have, then we proceed with these.

9) Finally, EMBO reports papers are accompanied online by A) a short (1-2 sentences) summary of the findings and their significance, B) 2-3 bullet points highlighting key results and C) a synopsis image that is 550x200-400 pixels large (width x height). You can either show a model or key data in the synopsis image. Please note that the size is rather small and that text needs to be readable at the final size. Please send us this information along with the revised manuscript.

Once you have made these minor revisions, please use the following link to submit your corrected manuscript:

https://embor.msubmit.net/cgi-bin/main.plex?el=A1Ij6BWs7A2COLj4J7A9ftdOZb8KzlZcuwDrLUfnAhtAAY

If all remaining corrections have been attended to, you will then receive an official decision letter from the journal accepting your manuscript for publication in the next available issue of EMBO reports. This letter will also include details of the further steps you need to take for the prompt inclusion of your manuscript in our next available issue.

Thank you for your contribution to EMBO reports.

REFEREE REPORTS

Referee #1:

The authors have addressed all my concerns. This is a nice paper.

Referee #3:

The authors successfully addressed the issues raised in the first round of the review and in its present form the manuscript meets EMBO Reports scientific merit.

The authors performed all minor editorial changes.

© European Molecular Biology Organization

EMBO PRESS

YOU MUST COMPLETE ALL CELLS WITH A PINK BACKGROUND

PLEASE NOTE THAT THIS CHECKLIST WILL BE PUBLISHED ALONGSIDE YOUR PAPER

Corresponding Author Name: David G McEwan	
Journal Submitted to: EMBO Reports	
Manuscript Number: EMBOR-2019-48412V2	

Reporting Checklist For Life Sciences Articles (Rev. June 2017)

This checklist is used to ensure good reporting standards and to improve the reproducibility of published results. These guidelines are consistent with the Principles and Guidelines for Reporting Preclinical Research issued by the NIH in 2014. Please follow the journal's authorship guidelines in preparing your manuscript.

A- Figures
1. Data
The data shown in figures should satisfy the following conditions:

- the data were obtained and processed according to the field's best practice and are presented to reflect the results of the experiments in an accurate and unbiased manner.
- figure panels include only data points, measurements or observations that can be compared to each other in a scientifically meaningful way.
- graphs include clearly labeled error bars for independent experiments and sample sizes. Unless justified, error bars should not be shown for technical replicates.
- if n< 5, the individual data points from each experiment should be plotted and any statistical test employed should be justified
- Source Data should be included to report the data underlying graphs. Please follow the guidelines set out in the author ship guidelines on Data Presentation.

2. Captions

Each figure caption should contain the following information, for each panel where they are relevant:

- a specification of the experimental system investigated (eg cell line, species name).
- the assay(s) and method(s) used to carry out the reported observations and measurements
- an explicit mention of the biological and chemical entity(ies) that are being measured.
- an explicit mention of the biological and chemical entity(ies) that are altered/varied/perturbed in a controlled manner.

- the exact sample size (n) for each experimental group/condition, given as a number, not a range;
- a description of the sample collection allowing the reader to understand whether the samples represent technical or biological replicates (including how many animals, litters, cultures, etc.).
- a statement of how many times the experiment shown was independently replicated in the laboratory.
- definitions of statistical methods and measures:
  - common tests, such as t-test (please specify whether paired vs. unpaired), simple χ2 tests, Wilcoxon and Mann-Whitney tests, can be unambiguously identified by name only, but more complex techniques should be described in the methods section;
  - are tests one-sided or two-sided?
  - are there adjustments for multiple comparisons?
  - exact statistical test results, e.g., P values = x but not P values < x;
  - definition of 'center values' as median or average;
  - definition of error bars as s.d. or s.e.m.

Any descriptions too long for the figure legend should be included in the methods section and/or with the source data.

In the pink boxes below, please ensure that the answers to the following questions are reported in the manuscript itself. Every question should be answered. If the question is not relevant to your research, please write NA (non applicable). We encourage you to include a specific subsection in the methods section for statistics, reagents, animal models and human subjects.

USEFUL LINKS FOR COMPLETING THIS FORM

http://www.antibodypedia.com
http://1degreebio.org
http://www.equator-network.org/reporting-guidelines/improving-bioscience-research-repo
http://grants.nih.gov/grants/olaw/olaw.htm
http://www.mrc.ac.uk/Ourresearch/Ethicsresearchguidance/Useofanimals/index.htm
http://ClinicalTrials.gov
http://www.consort-statement.org
http://www.consort-statement.org/checklists/view/32-consort/66-title
http://www.equator-network.org/reporting-guidelines/reporting-recommendations-for-tun
http://datadryad.org
http://figshare.com
http://www.ncbi.nlm.nih.gov/gap
http://www.ebi.ac.uk/ega
http://biomodels.net/
http://biomodels.net/miriam/
http://jjj.biochem.sun.ac.za
http://oba.od.nih.gov/biosecurity/biosecurity_documents.html
http://www.selectagents.gov/

B- Statistics and general methods

Please fill out these boxes (Do not worry if you cannot see all your text once you press return)

1.a. How was the sample size chosen to ensure adequate power to detect a pre-specified effect size?	Not applicable
1.b. For animal studies, include a statement about sample size estimate even if no statistical methods were used.	Not applicable
2. Describe inclusion/exclusion criteria if samples or animals were excluded from the analysis. Were the criteria pre-established?	Not applicable
3. Were any steps taken to minimize the effects of subjective bias when allocating animals/samples to treatment (e.g. randomization procedure)? If yes, please describe.	Not applicable
For animal studies, include a statement about randomization even if no randomization was used.	Not applicable
4.a. Were any steps taken to minimize the effects of subjective bias during group allocation or/and when assessing results (e.g. blinding of the investigator)? If yes please describe.	Not applicable
4.b. For animal studies, include a statement about blinding even if no blinding was done	Not applicable
5. For every figure, are statistical tests justified as appropriate?	Not applicable
Do the data meet the assumptions of the tests (e.g., normal distribution)? Describe any methods used to assess it.	Not applicable

Is there an estimate of variation within each group of data?	Not applicable
Is the variance similar between the groups that are being statistically compared?	Not applicable

C- Reagents

6. To show that antibodies were profiled for use in the system under study (assay and species), provide a citation, catalog number and/or clone number, supplementary information or reference to an antibody validation profile. e.g., Antibodypedia (see link list at top right), 1DegreeBio (see link list at top right).	included
7. Identify the source of cell lines and report if they were recently authenticated (e.g., by STR profiling) and tested for mycoplasma contamination.	included

* for all hyperlinks, please see the table at the top right of the document

D- Animal Models

8. Report species, strain, gender, age of animals and genetic modification status where applicable. Please detail housing and husbandry conditions and the source of animals.	Not applicable
9. For experiments involving live vertebrates, include a statement of compliance with ethical regulations and identify the committee(s) approving the experiments.	Not applicable
10. We recommend consulting the ARRIVE guidelines (see link list at top right) (PLoS Biol. 8(6), e1000412, 2010) to ensure that other relevant aspects of animal studies are adequately reported. See author guidelines, under 'Reporting Guidelines'. See also: NIH (see link list at top right) and MRC (see link list at top right) recommendations. Please confirm compliance.	Not applicable

E- Human Subjects

11. Identify the committee(s) approving the study protocol.	Not applicable
12. Include a statement confirming that informed consent was obtained from all subjects and that the experiments conformed to the principles set out in the WMA Declaration of Helsinki and the Department of Health and Human Services Belmont Report.	Not applicable
13. For publication of patient photos, include a statement confirming that consent to publish was obtained.	Not applicable
14. Report any restrictions on the availability (and/or on the use) of human data or samples.	Not applicable
15. Report the clinical trial registration number (at ClinicalTrials.gov or equivalent), where applicable.	Not applicable
16. For phase II and III randomized controlled trials, please refer to the CONSORT flow diagram (see link list at top right) and submit the CONSORT checklist (see link list at top right) with your submission. See author guidelines, under 'Reporting Guidelines'. Please confirm you have submitted this list.	Not applicable
17. For tumor marker prognostic studies, we recommend that you follow the REMARK reporting guidelines (see link list at top right). See author guidelines, under 'Reporting Guidelines'. Please confirm you have followed these guidelines.	Not applicable

F- Data Accessibility

18: Provide a "Data Availability" section at the end of the Materials & Methods, listing the accession codes for data generated in this study and deposited in a public database (e.g. RNA-Seq data: Gene Expression Omnibus GSE39462, Proteomics data: PRIDE PXD000208 etc.) Please refer to our author guidelines for 'Data Deposition'.<br><br>Data deposition in a public repository is mandatory for:<br>a. Protein, DNA and RNA sequences<br>b. Macromolecular structures<br>c. Crystallographic data for small molecules<br>d. Functional genomics data<br>e. Proteomics and molecular interactions	Sequences prived in material and methods fro guides used to generate knockouts and knock-ins.
19. Deposition is strongly recommended for any datasets that are central and integral to the study; please consider the journal's data policy. If no structured public repository exists for a given data type, we encourage the provision of datasets in the manuscript as a Supplementary Document (see author guidelines under 'Expanded View' or in unstructured repositories such as Dryad (see link list at top right) or Figshare (see link list at top right).	Not applicable
20. Access to human clinical and genomic datasets should be provided with as few restrictions as possible while respecting ethical obligations to the patients and relevant medical and legal issues. If practically possible and compatible with the individual consent agreement used in the study, such data should be deposited in one of the major public access-controlled repositories such as dbGAP (see link list at top right) or EGA (see link list at top right).	Not applicable
21. Computational models that are central and integral to a study should be shared without restrictions and provided in a machine-readable form. The relevant accession numbers or links should be provided. When possible, standardized format (SBML, CellML) should be used instead of scripts (e.g. MATLAB). Authors are strongly encouraged to follow the MIRIAM guidelines (see link list at top right) and deposit their model in a public database such as Biomodels (see link list at top right) or JWS Online (see link list at top right). If computer source code is provided with the paper, it should be deposited in a public repository or included in supplementary information.	Not applicable

G- Dual use research of concern

22. Could your study fall under dual use research restrictions? Please check biosecurity documents (see link list at top right) and list of select agents and toxins (APHIS/CDC) (see link list at top right). According to our biosecurity guidelines, provide a statement only if it could.	No
